# Gene editing of the multi-copy H2A.B gene and its importance for fertility

**DOI:** 10.1186/s13059-019-1633-3

**Published:** 2019-01-31

**Authors:** Nur Diana Anuar, Sebastian Kurscheid, Matt Field, Lei Zhang, Edward Rebar, Philip Gregory, Thierry Buchou, Josephine Bowles, Peter Koopman, David J. Tremethick, Tatiana A. Soboleva

**Affiliations:** 1 The John Curtin School of Medical Research, The Australian National University, Canberra, ACT 2601 Australia; 2 Present Address: James Cook University, PO Box 6811, Cairns, QLD 4870 Australia; 3 Sangamo Therapeutics, 501 Canal Blvd, Richmond, CA 94804 USA; 4 Present Address: bluebird bio, 60 Binney St, Cambridge, MA 02142 USA; 5 CNRS UMR 5309, Inserm U1209, Universite’ Grenoble Alpes, Institute for Advanced Biosciences, 38700 Grenoble, France; 6 Institute for Molecular Bioscience, The University of Queensland, Brisbane, QLD 4072 Australia

**Keywords:** Chromatin, Histone variants, H2A.B, RNA polymerase II, Pre-mRNA splicing, Splicing speckles, Genome editing, TALENs

## Abstract

**Background:**

Altering the biochemical makeup of chromatin by the incorporation of histone variants during development represents a key mechanism in regulating gene expression. The histone variant H2A.B, H2A.B.3 in mice, appeared late in evolution and is most highly expressed in the testis. In the mouse, it is encoded by three different genes. H2A.B expression is spatially and temporally regulated during spermatogenesis being most highly expressed in the haploid round spermatid stage. Active genes gain H2A.B where it directly interacts with polymerase II and RNA processing factors within splicing speckles. However, the importance of H2A.B for gene expression and fertility are unknown.

**Results:**

Here, we report the first mouse knockout of this histone variant and its effects on fertility, nuclear organization, and gene expression. In view of the controversy related to the generation of off-target mutations by gene editing approaches, we test the specificity of TALENs by disrupting the H2A.B multi-copy gene family using only one pair of TALENs. We show that TALENs do display a high level of specificity since no off-target mutations are detected by bioinformatics analyses of exome sequences obtained from three consecutive generations of knockout mice and by Sanger DNA sequencing. Male H2A.B.3 knockout mice are subfertile and display an increase in the proportion of abnormal sperm and clogged seminiferous tubules. Significantly, a loss of proper RNA Pol II targeting to distinct transcription–splicing territories and changes to pre-mRNA splicing are observed.

**Conclusion:**

We have produced the first H2A.B knockout mouse using the TALEN approach.

**Electronic supplementary material:**

The online version of this article (10.1186/s13059-019-1633-3) contains supplementary material, which is available to authorized users.

## Background

Histones and DNA form the core structure of chromatin, the nucleosome core, in which ~ 145 base pairs of DNA are wrapped around a histone octamer comprising of a (H3-H4)_2_ tetramer flanked by two H2A-H2B dimers. Importantly, the structure and the protein interaction surface of a nucleosome can be altered by the substitution of one or more of the major core histones with their variant forms to regulate gene expression and other DNA-dependent processes.

Despite being discovered over a decade ago [1], the in vivo importance of the mammalian histone H2A variant H2A.B remains unknown. H2A.B appeared late in evolution in mice (H2A.B.3) and humans (H2A.B), and it is predominantly expressed in the testis with low expression levels in the brain [2, 3]. In the testis, it is expressed from the pachytene stage of the prophase of meiosis until the haploid round spermatid stage where its expression peaks. The expression of H2A.B.3 is coincident with the highest level of transcription in the testis.

A striking in vitro feature of H2A.B/B.3 is their ability to completely unfold chromatin thus permitting high levels of RNA Pol II transcription [2, 3]. In the testis, ChIP-Seq and RNA-Seq experiments revealed that H2A.B.3 was incorporated both at the transcription start site and the gene body of a gene and this incorporation was positively correlated with gene expression. Consistent with these locations, ChIP-mass spec experiments revealed that H2A.B.3 directly interacts with the initiation and elongation forms of RNA Pol II and RNA processing factors [3]. Therefore, H2A.B may enhance transcription directly by decompacting chromatin and/or indirectly by recruiting RNA Pol II to an active gene. Most interestingly, the nuclei of round spermatids revealed a novel nuclear organization where large domains of H2A.B.3-containing chromatin exist, which colocalize with splicing speckles and the initiation and elongation forms of RNA Pol II indicating that active transcription occurs within splicing speckles [3]. Taken together, an attractive hypothesis is that RNA Pol II might be recruited to splicing speckles by H2A.B.3 [3] to facilitate high levels of transcription.

The availability of a H2A.B.3 knockout would therefore be an invaluable tool for investigating the importance of H2A.B in fertility and testing the above hypothesis. However, as the H2A.B.3 protein is expressed from three different genes, this is a technically challenging feat. Our aim was therefore to first devise the most efficient and specific strategy to inhibit the function of all three genes.

Previously, transcription activator-like effector nucleases (TALENs) were the choice to perform gene and genome editing [4, 5], but more recently this technology has been superseded by the CRISPR-Cas9 system [6]. However, while improvements are continually being made [7–9], one issue has been the extent of off-target mutants generated with the use of CRISPR-Cas9, which appears to be greater when compared with TALENs [10–14]. One attractive feature of TALENs, which enables a higher level of specificity, is that the Fok1 nuclease domain will only cleave DNA when dimerized, which occurs when the two TALE domains bind to DNA (on opposite strands) in close proximity to each other. Here, we used TALENs to genetically impair the function of all three H2A.B.3 encoding genes; moreover, we used only one pair of TALENs to do this. To our knowledge, this has not been done before. This KO H2A.B.3 mouse displayed a subfertile phenotype and revealed a correlation between the proper RNA Pol II targeting to distinct transcription–splicing territories and pre-mRNA splicing outcomes.

## Results

### The design, activity and specificity of TALENs

There are three H2A.B.3-encoding genes (H2Afb3, Gm14920, H2Afb2, which are > 92% identical) plus a pseudogene (Gm14904) all located on the X chromosome in the mouse (the pseudogene was present in the Ensemble release 57 but subsequently removed) (Additional file 1: Table S1). We confirmed the expression of the three H2A.B.3-encoding genes in the testis, and as expected, the pseudogene was not expressed (Additional file 2: Figure S1). In order to test the specificity of TALENs against the H2A.B.3 gene family, two groups of H2A.B.3-targeting TALENs were designed. The first group of 12 TALENs was designed to target only one gene, H2Afb3. The second group of 7 TALENs was designed to target all three H2A.B.3 genes (Additional file 3: Table S2).

The activity and specificity of the TALENs were tested by employing a Dual-Luciferase Single-Strand Annealing Assay (DLSSA) and by the Cel 1 cleavage assay. The TALEN plasmids were co-transfected into Neuro2a cells in pairs in various combinations (Additional file 4: Figure S2). The results for the first group showed that all TALEN pairs, in different combinations, had the highest activity for the H2Afb3 gene, with some TALEN pairs indeed only cleaving the H2Afb3 gene (e.g., 101408:101412). The second group of TALENs showed nuclease activity for all genes, with two pairs (101421:101422 and 101421:101423) having the highest activity. Cel 1 assays confirmed these findings (Additional file 5: Figure S3). These in vitro results demonstrate the high specificity of TALENs; some of the targeted DNA sequences of the first group, designed against the H2Afb3 gene, differed by only two to three base pairs compared to the second group, and yet specificity for only the H2Afb3 gene was achieved (Additional file 6: Figure S4). The TALEN pair 101421:101422 were used to knock out (KO) the function of all three H2A.B.3 genes in mice (Additional file 6: Figure S4).

Prior to creating the H2A.B.3 KO, we determined whether any off-target genomic locations, which have limited sequence homology to the TALEN pair 101421:101422, can be identified by using NCBI BLAST against the mouse reference genome mm10 built on the C57BL/6J strain. Even relaxing this BLAST search to only 8 bp out of the 15 bp DNA recognition sequence returned no matches (data not shown).

### Production of H2A.B.3 KO mice

In order to produce H2A.B.3 KO mice, FVB/NJArc mice (derived in Jackson Laboratories and sourced from Australian Animal Resource Centre (ARC), hence an extra suffix, JArc) were used. Capped, poly-adenylated, and purified in vitro transcribed mRNA was adjusted to 10 ng/μl for each TALEN pair (101421:101422) and injected into 532 fertilized one-cell embryos and cultured overnight to the two-cell stage. One hundred fifty-three surviving two-cell embryos that displayed normal morphology were implanted in pseudo pregnant females, resulting in the birth of 19 pups. Of these nineteen pups, nine contained TALEN-induced H2A.B.3 mutations (Table 1, italicized). Most interestingly, TALEN activity produced an all-or-nothing effect, i.e. for each of the nine founder pups, all three H2A.B.3 genes were mutated while for the ten unmodified pups, no gene copies of H2A.B.3 were modified (Table 1). Moreover, in female pups, both alleles of H2Afb3, Gm14920, and H2Afb2 were modified, and each H2A.B.3 gene contained a unique indel as the result of TALEN activity (identified by Sanger sequencing). Therefore, one pair of TALENs can edit six homologous alleles simultaneously.

**Table 1:** Genotype of all 19 pups born following TALEN injections

#	Pup ID	Genotype by Sanger sequencing		
		H2Afb3	Gm14920	H2Afb2
1	♀ L74-1	XX (wt)	XX (wt)	XX (wt)
2	♀ L74-2	XX (wt)	XX (wt)	XX (wt)
3	♀ L74-3	XX (wt)	XX (wt)	XX (wt)
4	♂ L74-4	XY (wt)	XY (wt)	XY (wt)
5	♂ L74-5*	*X* ^*∆5 + 286*^ *Y*	*X* ^*∆15*^ *Y*	*X* ^*∆17*^ *Y*
6	♀ L79-1	*X* ^*∆10*^ *X* ^*∆10*^	*X* ^*∆12*^ *X* ^*∆12*^ Chimera of b/∆/c	*X* ^*∆15*^ *X* ^*∆15*^ Chimera of c/∆/b
7	♀ L89-1*	*X* ^*∆22*^ *X* ^*∆160*^	*X* ^*∆5*^ *X* ^*∆17*^ Chimera c/∆/b)	*X* ^*∆5*^ *X* ^*∆5*^
8	♂ L89-2	*X* ^*∆65*^ *Y*	*X* ^*C/T, A/G*^ *Y*	X^∆42^ Y Chimera b/∆/c)
9	♂ L89-3	*X* ^*∆26*^ *Y* Chimera c/∆/a)	*X* ^*∆10*^ *Y* Chimera b/∆/c)	*X* ^*∆10*^ *Y* Chimera b/∆/c)
10	♀ L90-1	XX (wt)	XX (wt)	XX (wt)
11	♀ L90-2	XX (wt)	XX (wt)	XX (wt)
12	♀ L90-3*	*X* ^*∆28*^ *X* ^*∆5*^	*X* ^*∆10*^ *X* ^*∆10*^	*X* ^*∆64*^ *X* ^*∆64*^
13	♀ L90-4	*X* ^*∆15*^ *X* ^*∆10*^	No successful PCR amplification	No successful PCR amplification.
14	♀ L90-5	XX (wt)	XX (wt)	XX (wt)
15	♀ L90-6	*X* ^*∆17*^ *X* ^*∆17*^ Chimera c/∆/a)	*X* ^*∆5*^ *X* ^*∆5*^ Chimera b/∆/c)	*X* ^*∆5*^ *X* ^*∆5*^ Chimera c/b/∆/c)
16	♀ L90-7	XX (wt)	XX (wt)	XX (wt)
17	♂ L90-8	*X* ^*∆16*^ *Y*	*X* ^*∆17*^ *Y* Chimera c/∆/c/b)	No successful PCR amplification.
18	♂ L90-9	XY (wt)	XY (wt)	XY (wt)
19	♂ L90-10	XY (wt)	XY (wt)	XY (wt)

wt, wild type offspring; ∆n, deletion of n nucleotides; +n, insertion of n nucleotides. *Denotes mice that were used as founders to establish H2A.B.3 KO colonies. The nine Talen induced mutations are italicized

Most of the mutants carried small deletions. Interestingly, in two founders, L90-4 and L90-8, at least one of the H2A.B.3 genes (usually gm14920 or H2Afb2) was not amplifiable by PCR. However, when we used mixed primer pairs for PCR amplification (e.g., gm14920-forward and H2Afb2-reverse), we were able to detect an amplification product. 

Subsequent Sanger DNA sequencing demonstrated that a fusion between gm14920 and H2Afb2 had occurred (data not shown). Unexpectedly, when the founder L74-5 mouse, which only displayed the deletions and insertions described in Table 1, was bred with a wild type (wt) female, the resulting G1 progeny also displayed a chimera between the gm14920 and H2Afb2 genes (Additional file 7: Figure S5). Continued breeding from these chimeric G1 H2A.B.3^−/x^ females with wt males (up to four generations), revealed that these additional mutations were lost after G2, but maintained original founder mutations (H2Afb3 X^∆+286^, gm14290 X^∆15^, H2Afb2 X^∆17^). This can be explained by the fact that TALEN activity can be retained for several cell divisions following their injection into the two-cell stage, thus producing a mosaic genotype, as previously observed [15, 16]. Collectively, the G1 generation of the nine founders H2A.B.3 genetically modified mice produced 22/78 mice displaying mosaicism, but by G3 a pure non-mosaic mouse colony was derived.

### Approach to identify possible TALEN-induced off-target mutations

The strategy for the identification of possible TALEN-induced off-target mutations in H2A.B.3^−/y^ mice was based on the sequencing of exomes of three related non-mosaic H2A.B.3^−/y^ mice from three consecutive generations (G1-G3). This strategy avoids the problem observed in previous studies where unrelated mice were compared to assess off-target effects [12]. Each exome sample was sequenced using 100-bp paired-end reads on the Illumina HiSeq 2000 sequencer to a depth of 350–600× coverage, yielding between 129 × 10^6^ and 228 × 10^6^ reads per sample (Additional file 8: Table S3). The breeding began by crossing ♀L90-3 with ♂L74-5 to produce phenotype NM4-G1 H2Afb3 (X^∆5^Y), gm14920 (X^∆10^Y), and H2Afb2 (X^∆64^Y) mice. Subsequent crosses between genetically modified H2A.B.3 female mice and wt males produced NM4-G2 and G3 H2Afb3 (X^∆5^Y), gm14920 (X^∆10^Y), and H2Afb2 (X^∆64^Y) mice for exon sequencing (Additional file 9: Figure S6). By crossing mutant females with wt males, any true off-target mutations would be identified because they would be diluted by 50% after each generation. On the other hand, natural sequence variations due to mouse strain differences would not be diluted by breeding (see below). Sequenced mouse exomes were run through our in-house variant detection pipeline (see Methods) to detect single nucleotide variants (SNVs), small indels, and larger structural variants. Importantly, TALENs do not usually produce SNVs, thus this type of variant serves as an internal measure for mouse strain differences.

As expected, alignment with the mm10 genome identified a large number of SNVs (~ 1.7 × 10^6^ for G1 and G2, and ~ 0.69×10^6^ for G3) and indels (~ 1.7 × 10^5^ for G1 and G2, ~ 0.6 × 10^5^ for G3) (Additional file 10: Table S4); however, neither followed a dilution pattern but rather correlated with the original number of sequencing reads. To remove the effect of mouse strain differences, a filter for FVB/NJ was included into the alignment, which indeed greatly reduced the number of SNVs and indels (Additional file 11: Table S5). Again, the remaining SNVs and putative indels did not follow a dilution pattern following successive generations.

### Experimental validation showing that indels were incorrectly predicted

An additional analysis was conducted to separate heterozygous from homozygous indels [17]. As shown (Additional file 12: Table S6), after filtering against the FVB/NJ genome, 417 NM4-G1, 420 NM4-G2, and 325 NM4-G3 heterozygous (0/1 + 0/2 + 1/2) indels remained after alignment with the mm10 genome, but again did not display a 50% dilution pattern from G1-G3. To provide further evidence that these remaining indels are due to strain differences between FVB/NJArc and FVB/NJ mice, we interrogated the 19 indels common to all three NM4 G1-G3 mice (Additional file 13: Figure S7, the location of these putative indels are shown in Additional file 14: Table S7).

**Fig. 1:**
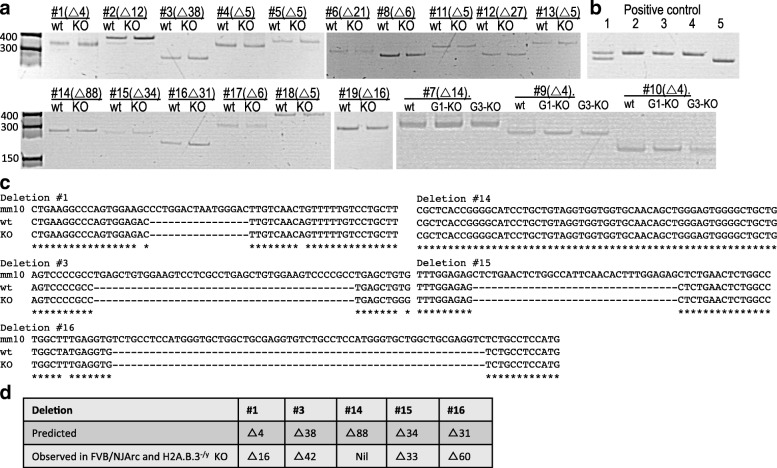
Interrogation of predicted off-target heterozygous deletions in H2A.B.3^−/y^ mice. **a** 300–400-bp regions surrounding 19 common predicted heterozygous deletions were amplified from gDNA of H2A.B.3^−/y^(KO) and FVB/NJArc in-house wt mice. PCR product sizes for wt and H2A.B.3^−/y^ were compared by resolving the products on a 7% polyacrylamide gel, side by side. The predicted deletion is indicated with △, followed by the number of nucleotides (nt) predicted to be deleted. **b** Positive controls showing that a deletion as small as 5 and 10 nt can be resolved on 7% polyacrylamide gel. Lane 1, a heterozygous △5 nt, lanes 2, 3, 4 wt.; lane 5, homozygous △10 nt. **c** 5 out of the 19 PCR products were sequenced by Sanger sequencing to show that FVB/NJArc and H2A.B.3^−/y^ mice have identical sequences but they are different from the mm10 reference genome. **d** Table showing that the prediction tools are often inaccurate at predicting the presence or the size of a putative deletion

We amplified ~ 300–450-bp regions spanning all 19 indels and compared them to the wt FVB/NJArc mice. The results for all 19 regions (Fig. 1a) clearly show that the amplified fragments were identical in size for the H2A.B.3^−/y^ and wt FVB/NJArc mice. Furthermore, we sequenced five of these putative indels, which confirmed that the H2A.B.3^−/y^ and wt mice are identical in the nucleotide sequence where the indels were predicted to be (Fig. 1b, c). Further, the computational prediction tools are often inaccurate, not only in predicting the size of a putative deletion but also in falsely predicting that a deletion exists (Fig. 1c, d). Overall, our results suggest that the predicted indels in our H2A.B.3^−/y^ are due to DNA sequence differences in the FVB/NJ mouse strains and computational errors in predicting indels. Finally, to detect structural variants in NM4-G1, NM4-G2, and NM4-G3, analyses using Pindel and Janda were performed. Only the three expected deletions corresponding to the three H2A.B.3 genes were detected: H2Afb3 (5 bp), gm14920 (10 bp), and a larger deletion of 64 bp in H2Afb2 (Additional file 15: Figure S8).

### H2A.B.3 KO mice are subfertile

**Fig. 2:**
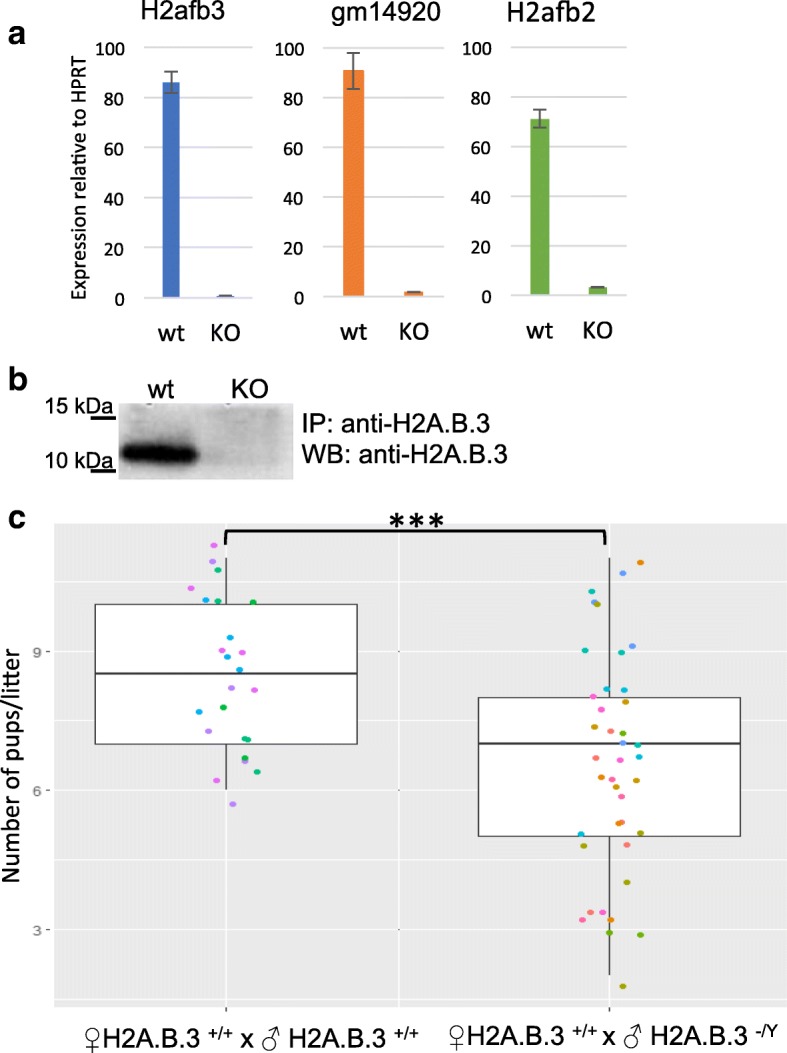
H2A.B.3 KO males have impaired fertility. **a** The mRNA level of each H2A.B.3 gene was determined by real-time quantitative PCR, relative to HPRT. Primers were designed to anneal within the TALEN-induced deletions. **b** Confirmation that H2A.B.3 protein is not produced in H2A.B.3^−/y^ mice. H2A.B was immunoprecipitated from total testis lysates followed by Western blotting using anti-H2A.B.3 polyclonal antibodies. **c** The breeding was conducted using age-matched wt females (3–6 months) and age-matched wt (*n* = 5) and H2A.B.3^−/y^ (*n* = 5) males. Total number of pups produced was 201 and 256 for wt and KO males, ***p value = ≤ 0.001 (ANOVA test)

Despite the fact that H2A.B.3-deficient male mice do not produce wt H2A.B.3 mRNA and protein (Fig. 2a, b), they can still reproduce and therefore spermatogenesis is not totally impaired. However, to investigate whether the loss of H2A.B.3 does affect fertility, we set up a breeding regime whereby age-matched male wt H2A.B.3 and H2A.B.3^−/y^ were mated with 3–6-month-old female mice and the litter sizes were recorded (Fig. 2c). The results showed that H2A.B.3^−/y^ mice were subfertile, producing significantly smaller litter sizes and thus demonstrating that H2A.B.3 is indeed required for fertility. Interestingly, some H2A.B.3^−/y^ mice were as fertile as wt mice while other H2A.B.3^−/y^ mice produced very small litters.

### H2A.B.3 KO mice have defective sperm

**Fig. 3:**
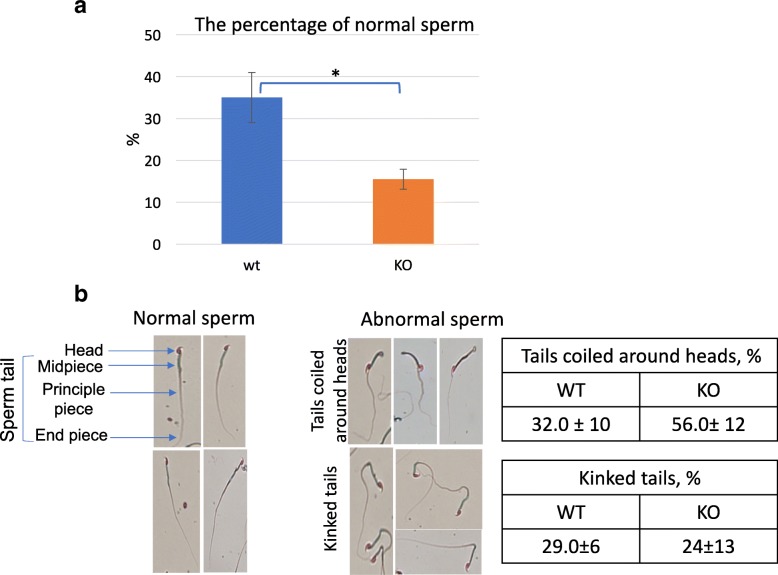
H2A.B.3 KO males have a higher percentage of abnormal sperm. **a** wt and H2A.B.3 KO mice were scored for abnormal sperm (*N* = 3). *p value <0.01 (T-test).  **b** The major abnormalities seen in both wt and H2A.B.3 KO sperm are those with a twisted tail around the head of the sperm and a kinked tail

To begin to understand the cellular causes for the observed subfertile phenotype, we first analyzed the quality of the sperm. In wt male mice, ~ 35% of sperm display no apparent abnormalities. On the other hand, only ~ 15% of sperm from H2A.B.3^−/y^ mice are normal (Fig. 3a). Two major sperm defects are observed in both wt and H2A.B.3 KO mice. Sperm that display a kinked tail or sperm with tails coiled around their heads (Fig. 3b). Compared to wt sperm, H2A.B.3^−/y^ sperm display a higher proportion of the latter defect (Fig. 3b). An overall decrease in viable sperm would obviously affect the total fertility rate [18]. Future in vitro fertilization experiments will further investigate the significance of these mutant sperm defects.

### Dynamics of TP1 chromatin incorporation and removal is altered in H2A.B.3 KO mice

Histone replacement by protamines, which requires transition proteins, is essential for the successful production of sperm. Interestingly, transition protein 1 (TP1)-deficient mice are subfertile and produce high proportion of defective sperm that appear to resemble the abnormal sperm observed from H2A.B.3^−/y^ mice (sperm with tails coiled around their heads) (Fig. 3) [18]. Therefore, we wondered, could the loss of H2A.B.3 affect the dynamics of TP1 incorporation?

**Fig. 4:**
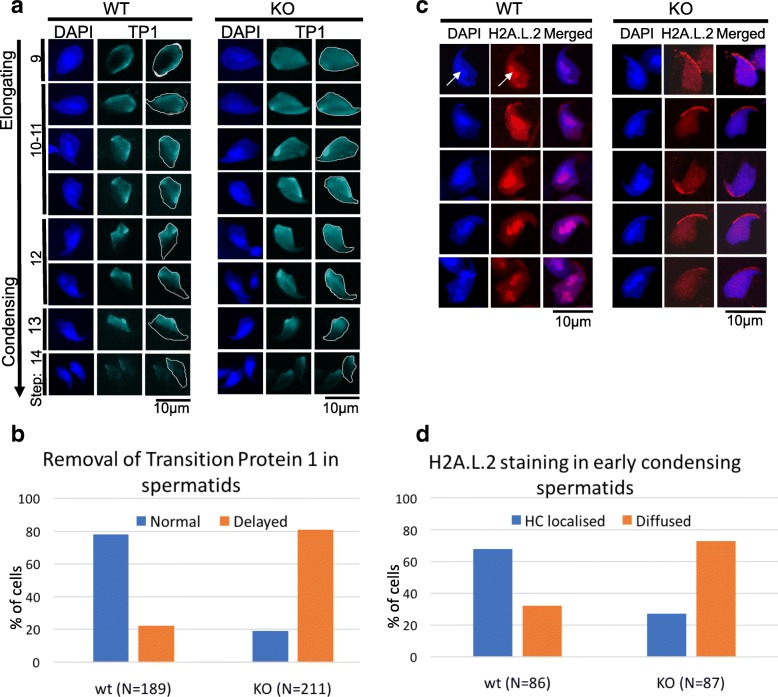
Characterization of the incorporation of TP1 and H2A.L.2 into spermatid chromatin in the absence of H2A.B.3. **a** Elongating/condensing spermatids immunostained with anti-TP1 antibodies (turquoise) and counterstained with DAPI (blue). The outlines of the spermatids nuclei are shown as a white trace in TP1 panels. **b** The quantification of the number of wild type and H2A.B.3^−/y^ step 10–12 spermatids with a delayed wave of TP1 release. **c** HA.L.2 localization in condensing wild type and H2A.B.3^−/y^ spermatid. White arrows point to pericentric heterochromatin. **d** The quantification of the number of wild type and H2A.B.3^−/y^ condensing spermatids with H2A.L.2 enriched in pericentric heterochromatin (HC)

TP1 begins to incorporate into chromatin in step 9 elongating spermatids and by step 14, is replaced by the protamines. In wt mice, the incorporation of TP1 occurs as a gradual wave across steps 9–11 spermatid nuclei with an apparent delayed incorporation into pericentric heterochromatin (the intense DAPI-stained regions) (Fig. 4a). Similarly, its removal also occurs as a wave-like process from steps 11–14. Most interestingly, H2A.B.3 KO spermatids display a different mode of TP1 incorporation and release from chromatin. In H2A.B.3 KO elongating spermatids, the incorporation of TP1 appears to be unregulated because already by step 9, TP1 is found throughout the nucleus including being present at pericentric heterochromatin (Fig. 4a, b). The wave of replacement of TP1 by protamines appears to be delayed, with TP1 still prominently present throughout the nucleus of step 10–12 elongating/condensing spermatid chromatin, whereas in the corresponding wt spermatids, it is not (Fig. 4a, b).

Transition proteins and the histone H2A variant H2A.L.2 are co-expressed, and recently, it was shown that the proper loading of TP1 was dependent upon the prior incorporation of H2A.L.2 [19]. We therefore investigated whether the loss of H2A.B.3 might affect the localization of H2A.L.2 in condensing spermatid nuclei where it is maximally expressed. As reported previously, in wt spermatids, H2A.L.2 is incorporated into all chromatin including pericentric chromatin (Fig. 4c) [19]. Strikingly, in KO spermatids, H2A.L.2 is no longer enriched in pericentric heterochromatin in the majority of condensing spermatids indicating that indeed the proper incorporation of H2A.L.2 is impaired (Fig. 4c, d). This defect in H2A.L.2 localization is not due to changes in its expression (data not shown). Taken together, it is plausible to suggest that this misincorporation of H2A.L.2 might contribute to the altered TP1 binding observed in H2A.B.3-deficient elongating/condensing spermatids.

### H2A.B.3 KO mice have clogged seminiferous tubules

**Fig. 5:**
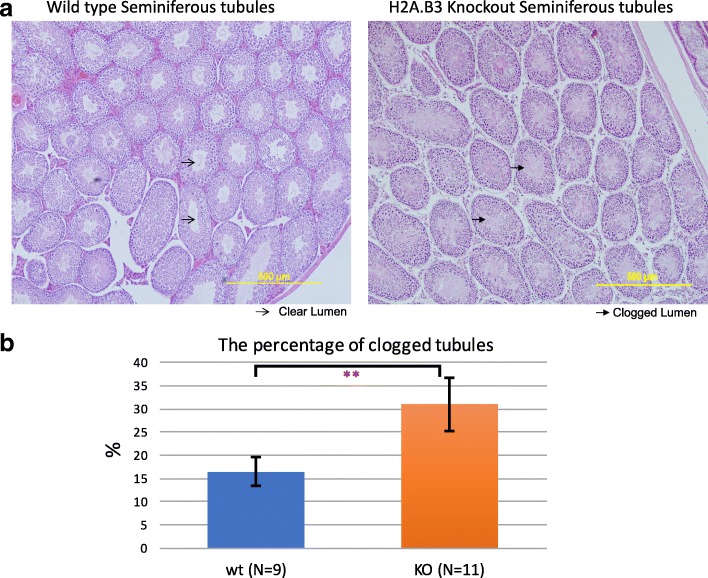
H2A.B.3 KO males have a higher percentage of clogged seminiferous tubules. **a** Image of hematoxylin and eosin-stained seminiferous tubule from wt and H2A.B.3^−/y^ mice. **b** Histogram plot of the percentage of a seminiferous tubule with a clogged lumen in H2A.B.3^−/y^ mice (*n* = 9) and their wt siblings (*n* = 11). ANOVA test, **p = 0.006547 (≤ 0.01)

Next, we compared seminiferous tubules of wt and H2A.B.3 KO mice to investigate if the site of sperm production and release is altered. Strikingly, while no defects are observed in seminiferous tubules themselves, a significant higher proportion of H2A.B.3^−/y^ testes display a clogged tubule phenotype (Fig. 5). During spermatogenesis, a considerable amount of cytoplasm is lost from maturing spermatids and released in the lumen. This material is subsequently cleared by Sertoli cells [2]. The presence of clogged lumen suggests that this process is impaired in H2A.B.3 KO testes, which may also contribute to the subfertile phenotype.

### Localization of RNA polymerase II within splicing speckles is perturbed in H2A.B.3 KO round spermatids

Previously, we observed a striking nuclear organization in round spermatids where distinct domains of H2A.B.3-containing chromatin colocalize with splicing speckles. Moreover, these H2A.B.3-splicing speckle domains appear to be transcriptionally active based on its colocalization with the initiation and elongation forms of RNA Pol II [3]. Further, given that H2A.B.3-containing nucleosomes directly interact with these forms of RNA Pol II [3], we investigated whether the loss of H2A.B affects (1) the formation of splicing speckles and/or (2) the localization of RNA Pol II to splicing speckles.

To investigate the first possibility, we used the splicing speckle marker, Y12, to identify splicing speckles in the H2A.B.3^−/y^ versus wt rounds spermatids. The Y12 immunostaining patterns show that the formation and morphology of splicing speckles, defined as distinct Y12 foci that colocalize with DAPI-depleted regions, was not altered in H2A.B.3^−/y^ round spermatids (Additional file 16: Figure S9).

**Fig. 6:**
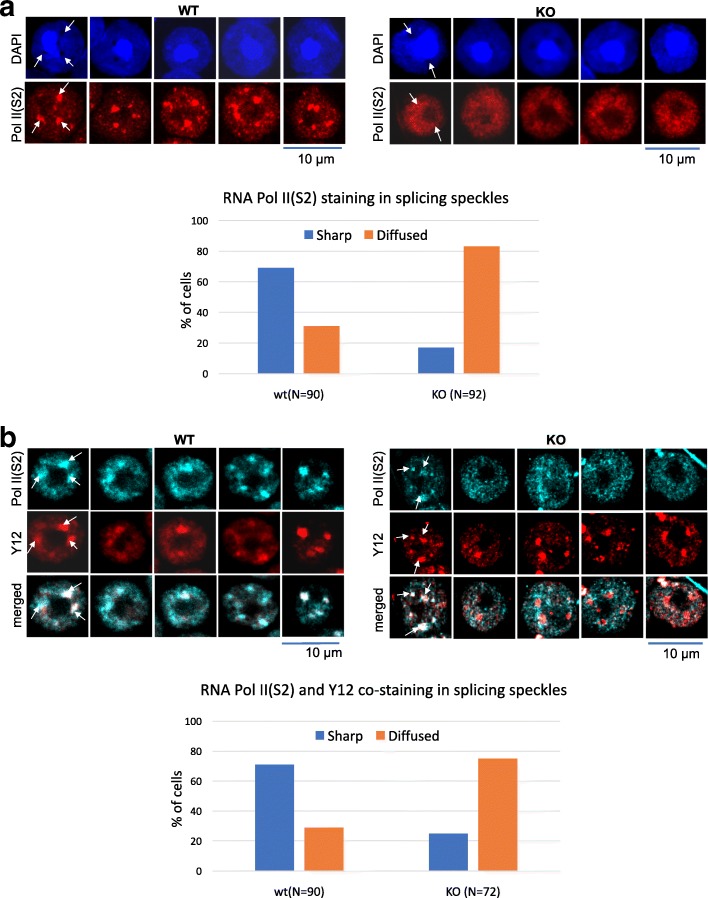
Localization of the active form of RNA polymerase II within splicing speckles is altered in H2A.B.3^−/y^ round spermatids. **a** Immunofluorescence staining of round spermatids from wt and H2A.B.3^−/y^ mice testis. Cells were indirectly labeled with anti-Pol II S2P antibodies and co-stained with DAPI. Scale bar is 10 μm. White arrows show accumulation of RNA Pol II S2P signal in splicing speckles of WT round spermatid cells. The signal is diffused in H2A.B.3^−/y^. Detailed quantification of RS in wt (*n* = 90) and H2A.B.3^−/y^ (*n* = 92) mice showing that majority of RS cells in H2A.B.3 KO mice have diffused Pol II S2 localization. **b** Immunofluorescence staining of round spermatids from wt and H2A.B.3^−/y^ mice testis. Cells were indirectly labeled with anti-Pol II S2P and Y2 (a splicing speckle marker) antibodies. Scale bar is 10 μm. White arrows show colocalization of RNA Pol II S2P and Y12 in splicing speckles of wt round spermatid cells. RNA Pol II S2P no longer co-stains with Y12 in the majority of round spermatids from H2A.B.3^−/y^ testes. Detailed quantification of RS in wt (*n* = 90) and H2A.B.3^−/y^ (*n* = 72) `

Next, we investigated whether the loss of H2A.B.3 affects the localization of Pol II to splicing speckles (Fig. 6). Immunofluorescent staining of round spermatids from wt and H2A.B.3^−/y^ mice using RNA Pol II S2P antibodies was performed. As expected, the immunostaining in the wt round spermatids produced strong RNA Pol II signals at splicing speckles (colocalizing with DAPI-depleted regions and with Y12, Fig. 6a, b). Strikingly, the distribution of RNA Pol II S2P in more than 70% of H2A.B.3^−/y^ round spermatids analyzed was significantly different—the immunostaining pattern was significantly more diffuse with the RNA Pol II signal no longer sharply focused into well-defined foci within DAPI-depleted regions (Fig. 6a) or in Y12 foci (Fig. 6b). Taken together, this supports the proposal that H2A.B.3 has a role in recruiting and/or stabilizing RNA Pol II at transcription–splicing speckles, but does not have a role in the formation of splicing speckles.

### The level of H3K4me3 increases in H2A.B.3 KO euchromatin

Next, we investigated whether the loss of H2A.B.3 had any impact on the abundance of post-translational modifications associated with heterochromatin (H3K9me2) or euchromatin (H3K4me3) (Additional file 17: Figure S10). Compared to wt round spermatids, there is no change in the abundance of H3K9me2 in euchromatin in H2A.B.3^−/y^ round spermatids (Additional file 17: Figure S10a). Strikingly, in the absence of H2A.B.3, there is a marked accumulation of H3K4me3 in euchromatin compared to wt round spermatids (Additional file 17: Figure S10b). On the other hand, we did not observe an increase in H3K4me2 or H4K8ac (data not shown).

To confirm this, we measured the fluorescence intensity of the H3K9me2 or H3K4me3 signal in the heterochromatic chromocenter (CC) and in the surrounding euchromatin (EU) and calculated their ratio (CC − B)/(EU − B), where B is background autofluorescence. This ratio would decrease if euchromatin contained more H3K9me2 or H3K4me3 than the heterochromatic chromocenter. We also scored two stages of round spermatid differentiation, the early round spermatid (ERS) and late round spermatid (LRS) stage. Indeed, the average (CC − B)/(EU − B) H3K4me3 ratio was smaller in H2A.B.3^−/y^ round spermatids compared to wt round spermatids whereas the ratio for H3K9me2 did not change significantly (Additional file 17: Figure S10). Recently, it was shown that the removal of the testis H2B histone variant TH2B induces compensatory mechanisms including lysine crotonylation and arginine methylation [19]. Given the existence of such redundant epigenetic mechanisms to ensure reproduction, it is attractive to speculate that this increase in H3K4me3 might be one mechanism that compensates for the loss of H2A.B.3.

### Effect of H2A.B.3 loss on transcription and splicing

Given that H2A.B.3 is incorporated into the chromatin of an active gene, we next performed a comprehensive differential gene expression analysis on purified round spermatids using high-throughput 75-bp paired-end sequencing of poly(A)-selected RNA (in triplicate but each mRNAseq library contained mRNA combined from two wt or H2A.B.3 KO mice). One notable initial observation from our analyses was the large variation in the RNA expression profile between different H2A.B.3 KO biological replicates compared to wt replicates, as indicated by a principal component analysis (Additional file 18: Figure S11a). Within this background of a large variation in gene expression between H2A.B.3 KO biological replicates, a differential gene expression volcano plot reveals that the expression of 86 genes are significantly altered in the absence of H2A.B.3 (Additional file 18: Figure S11b; Additional file 19: Table S8).

Given that H2A.B.3 can directly interact with RNA splicing factors on active genes [3] and that the loss of H2A.B.3 results in RNA Pol II no longer being localized at splicing speckles, we utilized the RNA-Seq data to determine whether alternative splicing is impacted by the loss of H2A.B.3. Similar to the RNA-Seq analysis, there was a large variation between individual biological repeats of H2A.B.3 KO mice but despite this variability 3278 small but significant differential splicing events where identified for a total of 1550 genes (Table 2, Additional file 20: Figure S12, Additional file 21: Table S9, Additional file 22: Table S10, Additional file 23: Table S11, Additional file 24: Table S12, Additional file 25: Table S13, Additional file 26: Table S14 Additional file 27: Table S15 and Additional file 28: Table S16). The most common observed alteration was a change in the relative expression of different mRNA isoforms (1940 events; differential transcript usage). Individual gene examples of differential transcript usage are *Fblim1* exon 6, *Slc37a3* exon 5, and *Traf7* exon 19 (Additional file 29: Figure S13). The analyses reveal other splicing changes (Table 2) including retained introns. An example of a gene displaying differential intron retention is *Styx* intron 3 (Additional file 29: Figure S13). Concerning the different splicing changes, Gene Ontology analyses of the different types of splicing defects reveals that no specific biological pathway is affected by the loss of H2A.B.3. This perhaps suggests that the loss of H2A.B.3 affects splicing in a non-specific manner. However, given the large variation in the RNA-Seq data between individual H2A.B.3 mice, this would make the identification of specific genes and/or pathways responsible for the observed fertility defects difficult to uncover.

**Table 2:** Differential splicing events when comparing H2A.B.3 KO with their wild type siblings

Total events*	3278
Differential transcript usage	1940
Alternative 5′ splice site	181
Alternative 3′ splice site	192
Alternative first exon	435
Alternative last exon	61
Use of mutually exclusive exons	50
Retained intron	53
Skipped exon	366

*The 3278 events were detected for a total of 1550 genes, which include 536 cases of genes where multiple local events affected the same gene

## Discussion

A major ongoing question is whether a RNA (e.g., CRISPR)- or protein (e.g., TALEN)-based mechanism will ultimately prove to be more specific for genome editing and this will be critical for future medical therapies. In this study, we have successfully applied the TALEN technology, whereby we used one pair of TALENs to specifically and simultaneously disrupt three gene copies of a gene family. Many human diseases are caused by gene copy number variations [20], and therefore, the use of a limited number of genome-editing enzymes might help in the future treatment of such diseases. The use of only one pair of TALENs, rather than multiple pairs, reduces the complexity of the TALEN approach (and hence the cost) and would reduce the likelihood of mosaicism and, most importantly, reduce the possibility of off-target mutations. Indeed, our rigorous exome analyses did not reveal any TALEN-induced off-target mutations. We also found that the computational tools used to predict putative SNVs and indels have limitations, and therefore wet-lab validation is required before any conclusions can be made about off-target mutations.

The TALEN approach did expose several interesting phenomena. Firstly, the simultaneous knockout of highly homologous H2A.B.3 genes leads to the formation of chimeras between these genes. As every chimera contained a small deletion, it is likely that terminal microhomology, a mechanism of NHEJ, was involved [21]. It is important to point out that other technologies that rely on NHEJ for inducing mutations, like CRISPR/Cas9 technology, will also likely produce chimeras between highly homologous genes. Several rounds of backcrossing can successfully eliminate such small-scale genomic rearrangements. Secondly, we observed an all-or-nothing effect whereby the TALENs either modified all three H2A.B.3 genes or modified none, which reduced the need for a more complex breeding and screening strategy to establish a H2A.B.3 KO colony.

Having successfully produced a H2A.B.3 knockout enabled us to begin to elucidate the importance of H2A.B.3 for fertility for the first time. H2A.B.3 KO mice are subfertile and consistent with this, our investigations reveal that no single major abnormality can be attributed to the loss of H2A.B.3 but instead several more subtle defects are observed, i.e., and increase in the proportion of defective sperm and clogged seminiferous tubules. Further, we do not know if the gene expression and RNA-splicing changes contribute to these defects. The fact that some H2A.B.3^−/y^ mice are as fertile as wt mice, whereas other male mice are extremely infertile hints at a possible compensatory mechanism (as observed for other histone variant knockouts [19]) that cannot always compensate for the loss of H2A.B.3.

It is attractive to speculate that the observed increase in H3K4me3 in H2A.B.3 KO round spermatid nuclei may be involved in such a compensatory mechanism, which might explain why our differential gene expression analysis did reveal a major change. On the other hand, the apparent large variation in the gene expression profile between individual H2A.B.3 KO testes suggests that, if H3K4me3 is involved in this compensation mechanism, its ability to compensate is stochastic, which could also explain why some H2A.B.3 KO male mice are fertile and others are less so.

It is interesting that TP1 KO mice display defective sperm that appear to resemble the abnormal sperm observed from H2A.B.3 KO mice, i.e., sperm with tails coiled around their heads [18]. This prompted us to examine TP1 chromatin loading and subsequent release and indeed, the timing of this process is altered in H2A.B.3 KO elongating/condensing spermatids. Furthermore, given that the proper loading of TP1 into euchromatin and heterochromatin requires prior assembly of H2A.L.2, we also investigated whether the incorporation of H2A.L.2 into chromatin was disrupted in the absence of H2A.B.3. The proper assembly of H2A.L.2 into chromatin was significantly impaired with a notable depletion in pericentric heterochromatin. This suggests that the misincorporation of H2A.L.2 might indeed contribute to the altered dynamics of TP1 binding and release. It is interesting that the function of one histone variant, H2A.B.3, can alter the function of another histone variant, H2A.L.2. The nature of this relationship will be the subject of further investigations.

A major finding is that in the absence of H2A.B.3, RNA Pol II no longer occupies the same territory as splicing speckles becoming more diffuse within round spermatid nuclei. It is therefore attractive to hypothesize that H2A.B.3 might have an important role in the recruitment and/or anchoring of RNA Pol II to active territories of transcription and splicing. This is consistent with previous mass spec data where it was shown that chromatin bound H2A.B.3 directly interacts with RNA Pol II [3]. Significantly, this loss of RNA Pol II localization is correlated with changes in pre-mRNA splicing.

## Conclusions

We have produced the first H2A.B knockout mouse using the TALEN approach. All three H2A.B.3 genes, were successfully genetically modified to inhibit their expression by using only one pair of TALENs. We also describe a robust approach to search for any off-targets and report several interesting TALEN-related phenomena applicable if other multi-copy genes are being considered for genome editing. H2A.B.3 KO male mice were subfertile and reveal several altered phenotypes that could account for this subfertility; an increase in the proportion of abnormal sperm and seminiferous tubules that are clogged. At the molecular level, TP1 chromatin loading and unloading is impaired. Most significantly, a loss of proper RNA Pol II targeting to distinct transcription–splicing territories and changes to pre-mRNA splicing were observed.

## Methods

### TALEN design

The TALENs were assembled using the method previously described [5, 22]. The TALEN target sites were searched using a computer script developed in Sangamo. The four most common repeat variable di-residue (RVD), NI, HD, NN, and NG, were used to recognize bases A, C, G, and T, respectively. The NK RVD was sometimes used to specify the 3′ half repeat T base. TALENs were generated by linking several pre-made TALE repeat blocks in the forms of monomer, dimer, trimer, or tetramer. The desired repeat blocks were PCR amplified using position-specific primers. The PCR products were pooled, digested with BsaI, and ligated into the TALEN expression vector, pVAXt-3Flag-NLS-TALE-Bsa3-C63-FokI, which had been linearized with the BsaI restriction enzyme. Ligations were transformed into *E. coli* competent cells, and single clones were picked and sequence verified.

### Dual-Luciferase Single-Strand Annealing Assay

The Dual-Luciferase Single-Strand Annealing Assay (DLSSA) is based on the Dual-Luciferase Reporter Assay System from Promega (Madison, WI). In the DLSSA, the firefly luciferase reporter gene is split into two inactive fragments with overlapping repeated sequences separated by stop codons and one of the three H2A.B.3 genes. Introduction of a double strand break in the H2A.B.3 gene by the TALEN pair initiates recombination between the flanking repeats by the single-strand annealing pathway and produces an active luciferase gene. Neuro2a cells (ATCC #CCL-131) were cultured in Dulbecco’s modified Eagle’s medium (DMEM, Cellgro Cat#: 10-013-CV) plus 10% FBS and 2 mM L-Glu (PSG optional antibiotic). One day before transfection, 20,000 cells per well were seeded into a 96-well plate. Cells were transiently transfected using Lipofectamine 2000 (Thermo Fisher Scientific) with four plasmids including a pair of TALENs, the SSA firefly luciferase reporter, and the internal control Renilla luciferase reporter (6.25 ng DNA for each component). The luciferase assay was performed 24 h post-transfection according to the manufacturer’s instructions. TALEN activity was measured as the ratio of the firefly and the Renilla luminescence units.

### Surveyor nuclease assay

The assay has been described in detail previously [5]. Briefly, mouse Neuro2A cells (2 × 10^5^) were seeded into a 96-well plate the day before transfection. The cells were then transfected with a pair of TALEN DNAs (400 ng of each) using Lipofectamine 2000 (Thermo Fisher Scientific). The genomic DNA was purified 48 h post-transfection. Gene-specific primer pairs were used to amplify each of the H2A gene variants. The primer sequences are H2Aafb3 forward (5′-CAGCAGAAAGCAGCCAAGTGG) and reverse (5′-GCAGGTCAGCCAAGAAGCA); Gm14920 forward (5′-GTACGGTACAAAGGGAG ATG) and reverse (5′-GAGCAGGTCAGCCAAGCAGAG); H2afb2 forward (5′-CAGGTCAGCAGAGAGCAATT) and reverse (5′-CTCCATACTGCTGTAGACCT); and pseudo Lap forward (5′-GTCAGCAGAATGCAGCCAAATAT) and reverse (5′-CAAGCCAGTAGCCAACATCAAG). The PCR products were treated with Surveyor Nuclease (Cel-1) and resolved on PAGE. The nuclease activity of TALENs was measured by quantifying the proportion of the cleaved DNA fragments.

### TALEN mRNA synthesis

Plasmids (in Sangamo backbone) were linearized with *XbaI* and the products were purified (PCR Cleanup kit, Qiagen). Capped and poly(A)-tailed mRNA transcripts were produced using a mMESSAGE mMACHINE T7 Ultra Transcription Kit (Life Technologies) following the manufacturer’s instructions. mRNA was purified using a MEGAclear kit (Life Technologies) and was eluted in RNase-free water, and single-use aliquots were frozen.

### Production of TALEN-mediated genetically modified mice

Prior to injection, mRNA was diluted to 10 ng/μl in filtered RNase-free microinjection buffer (10 mM Tris-HCl, pH 7.4, 0.25 mM EDTA). TALEN mRNA was delivered into the pronucleus of single-cell embryos (FVB), using standard techniques. Injected embryos were cultured overnight to the two-cell stage, then surgically transferred into oviducts pseudopregnant CD1 mice, using standard techniques.

### Analysis of potential founders and genotyping of successive generations

Transgenic founders with mutated H2A.B.3 genes were identified among live born mice by PCR (Additional file 30: Table S17) genotyping of ear notch tissue. Genomic DNA was extracted from ear punches using Quick Extract Solution (Epicentre) following the manufacturer’s instructions. The H2afb3, gm14920, and H2afb2 genes were amplified in 25 μl volume using the same gene-specific primers as for the cel1 assay. PCR mix (1× Platinum Taq buffer, 1.5 mM Mg2Cl; 0.2 mM dNTP; 0.2 μM of forward and reverse primer; 2 units of Platinum Taq polymerase (Thermo Fisher)) was amplified using 28 cycles of 95 °C 30 s denaturation, followed by 56–65 °C, 30 s annealing, and 72 °C, 4 min extension.

### Exome sequencing and bioinformatic analyses

The male mice that were chosen for exome sequencing were genotyped prior to sequencing to confirm that all three generations had the same H2A.B.3 genotype (H2Afb3 (X^∆5^Y), gm14920 (X^∆10^Y) and H2Afb2 (X^∆64^Y)). The purified gDNA was subjected to exome enrichment using the Agilent SureSelect^XT2^ All Exon Kit. The exome capture efficiency was uniformly high with approximately 45–50% of all DNA sequenced being exonic. Sequenced mouse exomes were analyzed using our in-house variant detection pipeline described in detail previously [23, 24]. In short, reads were aligned to the mouse reference genome mm10 using BWA [25] and sorted BAM alignment files were generated using SAMTools [26]. Candidate PCR duplicate reads were annotated using Picard software (http://broadinstitute.github.io/picard/), specifically the MarkDuplicate command. SNVs and small indels were called using SAMTools Mpileup [27], and variants were annotated using the Variant Effect Predictor [28]. To detect larger structural variants, both Pindel [29] and Janda (unpublished; https://sourceforge.net/projects/janda/) were utilized. To reduce the number of total variants called, variants specific to the FVBN mouse strain were downloaded from the Sanger Institute ftp site (ftp://ftp-mouse.sanger.ac.uk/REL-1206-FVBNJ/). As these variants were originally reported relative to reference genome mm9, the UCSC liftOver tool (https://genome.ucsc.edu/cgi-bin/hgLiftOver) was used to map the FVBN variants to mm10. These FVBN-specific variants were annotated and not considered for further off-target analysis.

For the detection of INDELs in the TALEN mouse exome sequencing data, we followed the GATK recommendations “Best Practices for Germline SNP & Indel Discovery in Whole Genome and Exome Sequence” [17]. Briefly, the alignment files in BAM format were realigned and base re-calibration was performed. The cleaned alignments of all sequenced mouse exomes were then used as input for the “HaplotypeCaller” program resulting in raw variants in VCF format, containing both SNVs and INDELs. We then performed hard filtering of the called variants to only include INDELS and applied a quality cut-off score of 100 to include only high-confidence INDEL calls. In order to identify potential TALEN off-targets, we inspected the frequency of INDELs in each generation of TALEN mice, expecting a “dilution” effect due to back-crosses.

In order to search the mm10 reference genome for potential off-target sites, we used the in silico PCR function of the UCSC Genome Browser at http://genome.ucsc.edu/cgi-bin/hgPcr [30]. We used the TALEN target sequences as forward and reverse primers, and relaxed the minimum perfect match parameter to eight nucleotides (nt) and constrained the maximum product size to 100 bp.

### Experimental testing of putative indels

To interrogate whether the 19 putative indels are present in NM4 G1-G3 mice, the gDNA of wild type FVB/NJArc in-house mice and NM4 G1-G3 KO mice were amplified with primers surrounding the predicted indels (listed in Additional file 31: Table S18). The PCR products were analyzed by electrophoresis (7% acrylamide gel) and by Sanger sequencing.

### Immunohistochemistry and testis surface spread preparations

The testes from 30-day-old mice (82% of tubules contain round spermatids at this stage) were removed and subjected to immumohistochemistry and DAPI staining as previously described [3].

### Purification of pure round spermatids from adult mouse testis

The purification was performed exactly as described elsewhere [31]. Briefly, one or two male mice were used per purification. The animals, aged between 8 and 10 weeks, were sacrificed, testes extracted, and albuginea removed. The seminiferous tubules were digested for 15 min at 35 °C with 1 mg/ml of collagenase in PBS, supplemented with lactate and glucose. The cells were washed and further dissociation was performed by vigorous pipetting for 10 min on ice. The cells were twice filtered through a 100-μm filter and loaded onto the 2–4% BSA gradient (in 360 ml). The sedimentation of cells was allowed to occur for 75 min. The cells were collected into 10-ml fractions, and each fraction was analyzed under the phase-contrast microscope. The fractions that contained only round spermatids were pooled together and used for mRNA purification.

### RNA-Seq

Round spermatids were purified from six H2A.B.3 KO and six wt males. Three biological replicates for each genotype were obtained by merging cells from two individuals. 4 × 10^6^ cells for each replica were used for RNA purification by Trizol. Prior to purification, ERCC RNA spike-in Mix 1 (diluted 1:10) was added to each sample that was already lysed in Trizol. The addition of spike-in control was done at this stage to control for RNA loss during the purification procedure. Purified RNA was resuspended in 50 μl of sterile MQ water. Fifteen microliters of each sample was subjected to Turbo DNase treatment and 3 μl of DNase-treated RNA was used for TruSeq Stranded mRNA LT Illumina library preparation. Samples were sequenced using 75-bp paired-end sequencing on a NextSeq500 platform. Data processing and analysis were as previously described by us [32]. Differential splicing analyses employed the SUPPA2 method, which takes into account biological variability [33]. Primers used to examine the differential splicing events for individual genes are the following:

- Fblim1 FW GTCTGTGGTTTCTGTCACAAG
- Fblim1 REV GGTAGCAGGGTTCGCACAG
- Slc37a3 FW GGACTCTGCTAGGCTTCATC
- Slc37a3 REV CTATACCCAACAAGGGACCC
- Styx FW CATTCAGCAATCACTCCTGC
- Styx REV GGTTTTCTTAATGTTAGTAGGAG
- Traf7 FW GTCTGGAGTATGGACAACATG
- Traf7 REV CTTCACGGTGCTATCCACAG

### Antibodies used in this study

Antibodies used in this study were rabbit polyclonal anti-TP1 (ab73135, IF 1:100), rabbit serum anti-H2A.L.2 (gift from S Khochbin, IF 1:50), rabbit polyclonal anti-Pol II S2 (ab5095, IF 1:100), mouse monoclonal anti-Y12 (gift from B Westman, IF 1:200), rabbit polyclonal anti-H3K4me3 (ab8580, IF 1:100), and mouse monoclonal anti-H3K9me2 (ab1220, IF 1:100).

## Additional files

Additional file 1: **Table S1.** The genomic coordinates of H2A.B.3 coding genes. The degree of homology between the genes is indicated as % Identity. (PDF 49 kb)

Additional file 2: **Figure S1.** The relative expression of H2A.B.3 gene family members in the testis of FVB/NJArc mice. Gene expression was assessed relative to mHPRT using qPCR. (PDF 86 kb)

Additional file 3: **Table S2.** List of TALENs designed to target H2A.B.3 genes. (PDF 51 kb)

Additional file 4: **Figure S2.** TALEN pair specificity tested by the DLLS assay. TALEN pairs designed to target the H2Afb3 gene only (Group 1) or all three H2A.B.3 genes (Group 2). One day before transfection, 20,000 cells per well were seeded into a 96-well plate. Cells were transiently transfected with four plasmids including a pair of TALENs, the SSA firefly luciferase reporter, and the internal control Renilla luciferase reporter. The luciferase assay was performed 24 h post-transfection. The pair 101421:101422 was used to create the H2A.B.3 KO mouse. (PDF 435 kb)

Additional file 5: **Figure S3.** Cel 1 assays. Neuro2a cells were transiently transfected with various TALEN pairs and extracted genomic DNA was amplified by PCR with gene-specific primers and subjected to Cel1 digestion. TALEN activity is represented by the % cleaved (% genomic mutation, GM) shown below each lane. Cleaved DNA products (arrow heads), uncut DNA (arrow). TALEN pair 101421:101422, highlighted in red, showed the highest activity for all three H2A.B.3 genes. 1,2,3 and P denotes H2Afb3, gm14920, H2Afb2 and pseudo H2A.B.3 genes, respectively. Two biological replicates are shown. Empty GFP-vector was used as a negative control. (PDF 1001 kb)

Additional file 6: **Figure S4.** TALEN-targeted DNA sequences. The alignment of H2A.B.3 genes and the pseudogene. TALENs specific to H2afb3 are in blue and those common for all H2A.B3 genes are in red. (PDF 58 kb)

Additional file 7: **Figure S5.** An example of chimeras formed between gm14920 and H2afb2 in some G1 mice produced by crossing founder L74-5 with a wt. female. (a) genes gm14920 and H2afb2 are not amplified with gene-specific primers in pups #6 and #7, respectively but is amplified in a pup #5. (b) amplification with gm14920-Fw and H2afb2-Rev primers shows the chimeric product in pups #6 and #7. (c) A schematic diagram showing that a chimera was formed between gm14920 and H2afb2 genes with a small deletion between these fused genes suggesting that NHEJ mechanisms were involved. Fw, forward primer; Rev., reverse primer; number; L, DNA ladder. (PDF 198 kb)

Additional file 8: **Table S3.** The exome sequencing coverage. Three consecutive generations of mice from the NM4 H2A.B.3 KO colony were sequenced using paired-end sequencing with a 100 bp read length. (PDF 48 kb)

Additional file 9: **Figure S6.** TALEN-induced deletions of the three H2A.B.3 genes. (PDF 92 kb)

Additional file 10: **Table S4.** Putative SNVs and Indels identified in H2A.B.3 KO mice using the mm10 mouse genome as a reference. (PDF 48 kb)

Additional file 11: **Table S5.** Putative SNVs and Indels identified in H2A.B.3 KO mice after applying the FVB/NJ strain filter. (PDF 48 kb)

Additional file 12: **Table S6.** Predicted putative homozygous and heterozygous polymorphic variants (off-target deletions) in three consecutive generations of H2A.B.3^−/y^ mice. Exome data was referenced to mm10 genome, followed by filtering of FVB/N-specific variants and analyzed for genome polymorphism using Pindel tool. Homozygous polymorphisms: no polymorphic alleles (0/0), both alleles differ from reference (1/1), and both alleles differ from reference and from 1/1 (2/2). Heterozygous polymorphisms: one allele differs from the reference genome (0/1), one allele defers from the reference genome and from 0/1 (0/2), both alleles differ from the reference genome and from each other (1/2). No call, not assigned to any type. (PDF 54 kb)

Additional file 13: **Figure S7.** Computational analysis predicted 19 putative heterozygous deletions shared between all 3 generations of H2A.B.3^−/y^ KO mice. If TALENs introduced off-target mutations in G0 founder mice, then those mutations would be inherited by their progeny as heterozygous. (PDF 83 kb)

Additional file 14: **Table S7.** Nineteen common putative heterozygous polymorphisms identified in all three generations of H2A.B.3^−/y^ mice. (PDF 72 kb)

Additional file 15: **Figure S8.** Exome sequencing reveals all the three expected mutations in H2A.B.3 genes. The screen shots are the output from SVs call by Pindel for all H2A.B.3 genes. The orange border box indicates the deleted region detected for each gene by SAMtools. (PDF 390 kb)

Additional file 16: **Figure S9.** Splicing speckle morphology is not affected by the absence of H2A.B.3. Round spermatids from wt (a) and H2A.B.3^−/y^ (b) were indirectly immunostained with anti-Y12 antibody and counterstained with DAPI. Scale bar is 10 μm. White arrows show accumulation of Y12 signal in DAPI-depleted regions. The immunostaining pattern showed no difference in Y12 localization. (PDF 1236 kb)

Additional file 17: **Figure S10.** H3K4me3 accumulates in euchromatin of H2A.B.3 KO round spermatids. Early round spermatids (ERS) and late round spermatids (LRS) were immunostained with H3K9me2 (panel a—red) and H3K4me3 (panel b—red) and Lectin PNA (green, a marker for acrosome to determine the stages of RS cells). DNA was co-stained with DAPI (blue). Scale bar is 10 μm. The quantification of H3K9me2 and H3K4me3 was based on the ratio of (CC − B)/(EU − B). *** ps0.001, ANOVA test. (PDF 1323 kb)

Additional file 18: **Figure S11.** Differentially expressed genes between H2A.B.3KO and wt round spermatids. (a) Principal component analysis of three sequenced H2A.B.3KO and wt mRNAseq libraries (each library combined mRNA from two wt or two H2A.B.3 KO mice). (b) Log_2_-changes in gene expression are plotted against significance (−log_10_-FDR). The plot illustrates in red those genes (Additional file 23: Table S11) whose expression is significantly altered in the absence of H2A.B.3. (PDF 201 kb)

Additional file 19: **Table S8.** Results of the differential RNA-Seq gene expression analysis comparing wt with H2A.B.3 KO round spermatids. (CSV 22 kb)

Additional file 20: **Figure S12.** Loss of H2A.B.3 affects pre-mRNA splicing. The analysis is based on SUPPA2. Illustrated is the distribution of altered splicing events by plotting the difference of percentage-spliced-in (PSI, the ratio between reads for included or excluded exons) versus transcript abundance of the transcripts associated with the splicing event (TPM) for two different conditions. The light gray dots correspond to (ΔPSI, TPM) values based on pairwise comparisons of WT versus H2A.B.3 KO replicates (i.e. three pairwise comparisons for the three biological replicates of WT versus KO) and TPM values averaged across replicates for each condition. The dark gray dots correspond to (ΔPSI, TPM) values after averaging PSI and TPM values across replicates for each condition. The dark gray dots show the difference in mean (averaged across replicates) PSI values and mean (averaged across replicates and conditions) TPM values. The blue dots represent events based on the replicate-averaged PSI values (the dark gray dots) that are statistically significant at an FDR of 5% (these numbers are shown in Table 2). (PDF 919 kb)

Additional file 21: **Table S9.** Results of the differential splicing analysis showing the differential transcript usage comparing wt with H2A.B.3 KO round spermatids. (XLSX 196 kb)

Additional file 22: **Table S10.** Results of the differential splicing analysis showing alternative 5′ splice site usage comparing wt with H2A.B.3 KO round spermatids. (XLSX 31 kb)

Additional file 23: **Table S11.** Results of the differential splicing analysis showing alternative 3′ splice site usage comparing wt with H2A.B.3 KO round spermatids. (XLSX 32 kb)

Additional file 24: **Table S12.** Results of the differential splicing analysis showing alternative first exon usage comparing wt with H2A.B.3 KO round spermatids. (XLSX 90 kb)

Additional file 25: **Table S13.** Results of the differential splicing analysis showing alternative last exon usage comparing wt with H2A.B.3 KO round spermatids. (XLSX 18 kb)

Additional file 26: **Table S14.** Results of the differential splicing analysis showing the use of mutually exclusive exons comparing wt with H2A.B.3 KO round spermatids. (XLSX 15 kb)

Additional file 27: **Table S15.** Results of the differential splicing analysis showing the number of retained introns comparing wt with H2A.B.3 KO round spermatids. (XLSX 15 kb)

Additional file 28: **Table S16.** Results of the differential splicing analysis showing the number of skipped exons comparing wt with H2A.B.3 KO round spermatids. (XLSX 63 kb)

Additional file 29: **Figure S13. **Differential splicing events. The differential splicing events for specific genes between wild type and H2A.B.3 KO identified by SUPPA analysis were visualized in the IGV browser. Representative images are shown for Fblim1 exon 6 (a; differential transcript usage), Styx intron 3 (b; retained intron), Slc37a3 exon 5 (d; differential transcript usage) and Traf7 exon 19 (e; differential transcript usage). These individual examples of differential splicing events were confirmed by qPCR (c and f). Purified round spermatids from four biological replicas of wild type and H2A.B.3 KO mice were used. β-actin was used as the internal control. Error bars represent standard deviation. Student T-test was used to calculate *p* values (** *p* < 0.01; ***, *p* < 0.001). (PDF 419 kb)

Additional file 30: **Table S17.** PCR primers for genotyping of H2A.B.3 encoding genes in founders and the H2A.B3KO colonies. (PDF 53 kb)

Additional file 31: **Table S18.** List of primers to amplify 19 putative Indels from TALEN mutant mice. Symbols: ∆, deletion. (PDF 72 kb)
