# Supplementary material for: Gene editing of the multi-copy H2A.B gene and its importance for fertility
Source: Genome Biol. 2019 Jan 31;20:23. doi: 10.1186/s13059-019-1633-3 (PMC6357441; doi:10.1186/s13059-019-1633-3)
Supplement: Supplementary file 1 — Table S1. The genomic coordinates of H2A.B.3 coding genes. The degree of homology between the genes is indicated as % Identity. (PDF 49 kb) [file 13059_2019_1633_MOESM1_ESM.pdf]

Gene names/length	Strand	Chromosome	Start	End	% Identity
H2afb3, 348bp ENSMUSG00000083616	-	X	117426357	117426704	100
Gm14920, 348bp ENSMUSG00000067441	+	X	114128366	114128713	98.28
H2afb2, 349bp EG624153	+	X	113794787	113795134	92.55
Gm14904(Pseudo), 345bp ENSMUSG00000084053	+	X	113311798	113312139	87.83

Table S1.
