# Supplementary figures and images for: Gene editing of the multi-copy H2A.B gene and its importance for fertility
Source: Genome Biol. 2019 Jan 31;20:23. doi: 10.1186/s13059-019-1633-3 (PMC6357441; doi:10.1186/s13059-019-1633-3)

Figure S1

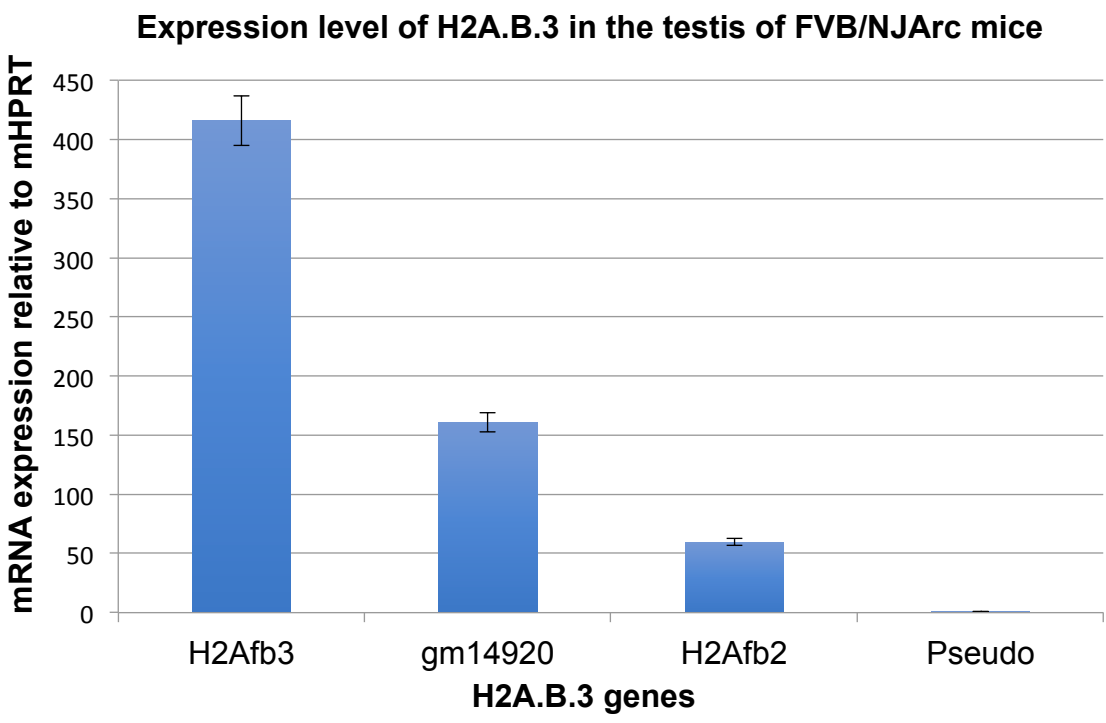

Supplement: Supplementary file 2 — Figure S1. The relative expression of H2A.B.3 gene family members in the testis of FVB/NJArc mice. Gene expression was assessed relative to mHPRT using qPCR. (PDF 86 kb) [file 13059_2019_1633_MOESM2_ESM.pdf]

Figure S2

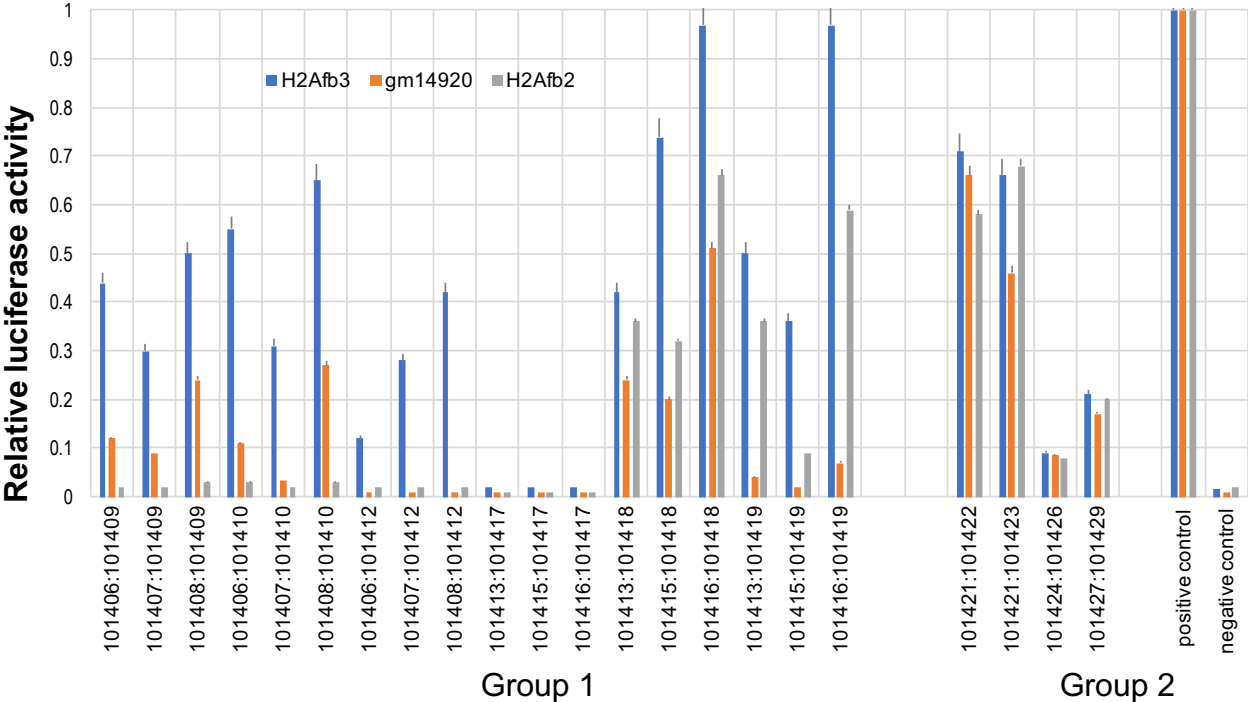

Supplement: Supplementary file 4 — Figure S2. TALEN pair specificity tested by the DLLS assay. TALEN pairs designed to target the H2Afb3 gene only (Group 1) or all three H2A.B.3 genes (Group 2). One day before transfection, 20,000 cells per well were seeded into a 96-well plate. Cells were transiently transfected with four plasmids including a pair of TALENs, the SSA firefly luciferase reporter, and the internal control Renilla luciferase reporter. The luciferase assay was performed 24 h post-transfection. The pair 101421:101422 was used to create the H2A.B.3 KO mouse. (PDF 435 kb) [file 13059_2019_1633_MOESM4_ESM.pdf]

Figure S3

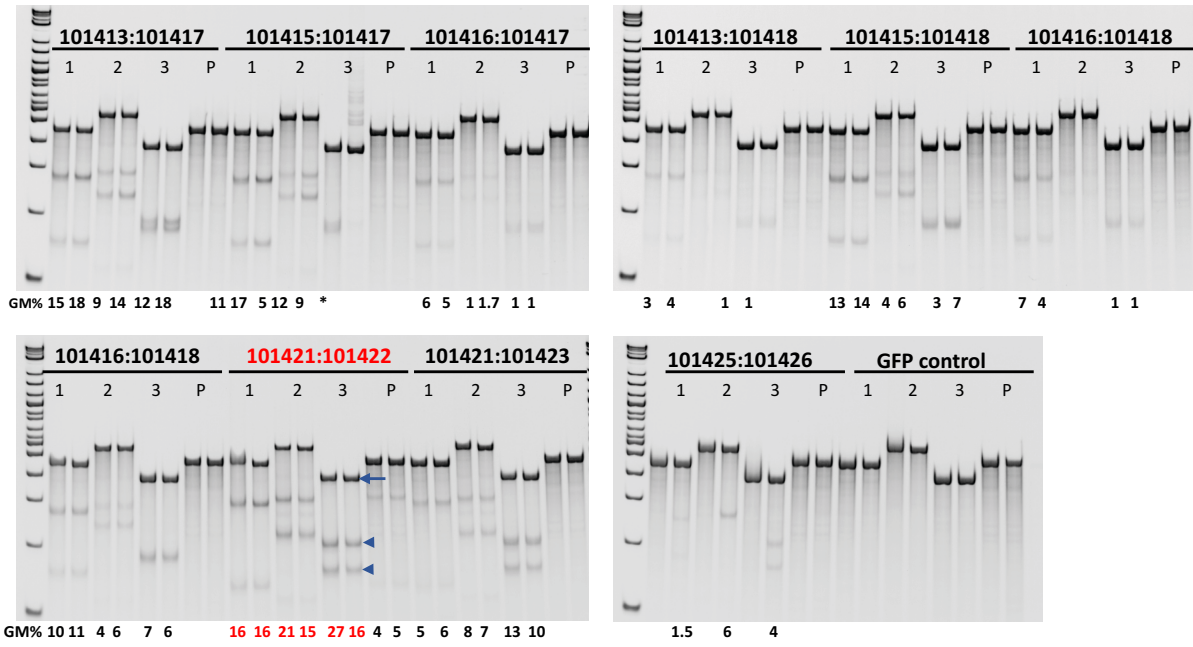

Supplement: Supplementary file 5 — Figure S3. Cel 1 assays. Neuro2a cells were transiently transfected with various TALEN pairs and extracted genomic DNA was amplified by PCR with gene-specific primers and subjected to Cel1 digestion. TALEN activity is represented by the % cleaved (% genomic mutation, GM) shown below each lane. Cleaved DNA products (arrow heads), uncut DNA (arrow). TALEN pair 101421:101422, highlighted in red, showed the highest activity for all three H2A.B.3 genes. 1,2,3 and P denotes H2Afb3, gm14920, H2Afb2 and pseudo H2A.B.3 genes, respectively. Two biological replicates are shown. Empty GFP-vector was used as a negative control. (PDF 1001 kb) [file 13059_2019_1633_MOESM5_ESM.pdf]

Figure S5

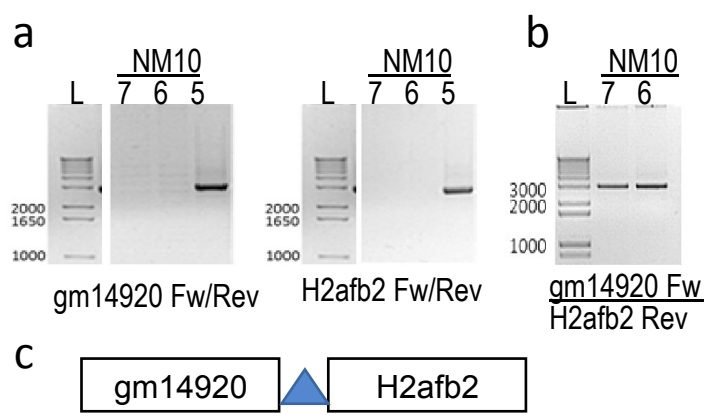

Supplement: Supplementary file 7 — Figure S5. An example of chimeras formed between gm14920 and H2afb2 in some G1 mice produced by crossing founder L74-5 with a wt. female. (a) genes gm14920 and H2afb2 are not amplified with gene-specific primers in pups #6 and #7, respectively but is amplified in a pup #5. (b) amplification with gm14920-Fw and H2afb2-Rev primers shows the chimeric product in pups #6 and #7. (c) A schematic diagram showing that a chimera was formed between gm14920 and H2afb2 genes with a small deletion between these fused genes suggesting that NHEJ mechanisms were involved. Fw, forward primer; Rev., reverse primer; number; L, DNA ladder. (PDF 198 kb) [file 13059_2019_1633_MOESM7_ESM.pdf]

Figure S7

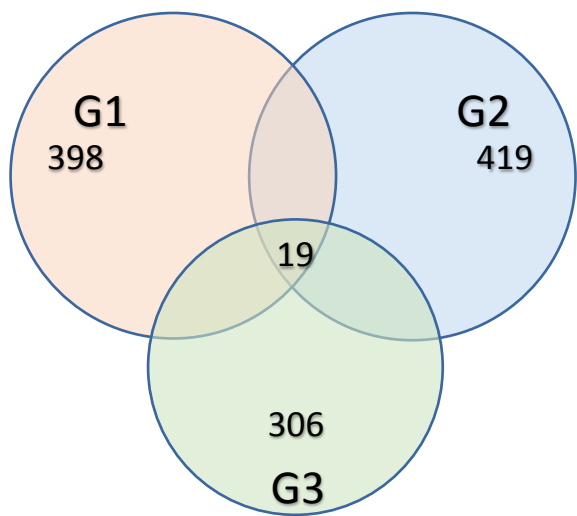

Supplement: Supplementary file 13 — Figure S7. Computational analysis predicted 19 putative heterozygous deletions shared between all 3 generations of H2A.B.3−/y KO mice. If TALENs introduced off-target mutations in G0 founder mice, then those mutations would be inherited by their progeny as heterozygous. (PDF 83 kb) [file 13059_2019_1633_MOESM13_ESM.pdf]

Figure S8

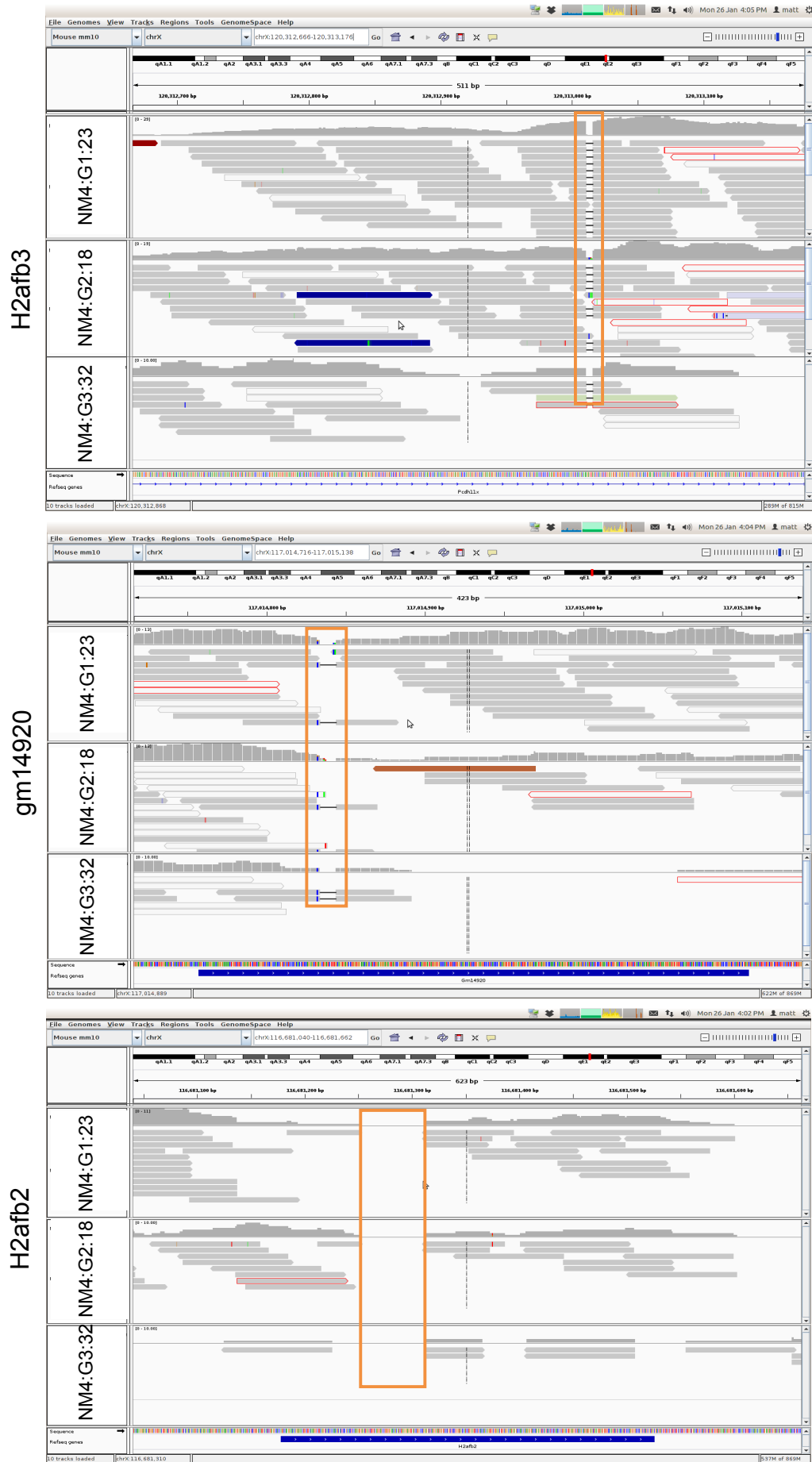

Supplement: Supplementary file 15 — Figure S8. Exome sequencing reveals all the three expected mutations in H2A.B.3 genes. The screen shots are the output from SVs call by Pindel for all H2A.B.3 genes. The orange border box indicates the deleted region detected for each gene by SAMtools. (PDF 390 kb) [file 13059_2019_1633_MOESM15_ESM.pdf]

Figure S9

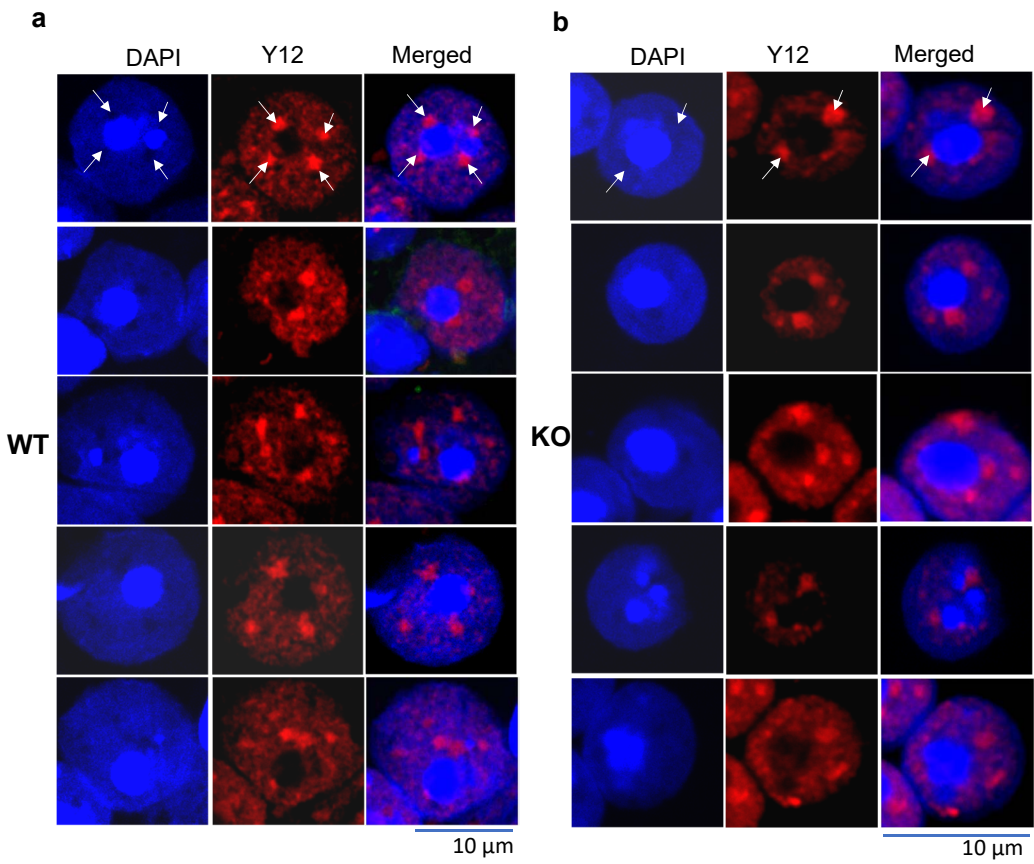

Supplement: Supplementary file 16 — Figure S9. Splicing speckle morphology is not affected by the absence of H2A.B.3. Round spermatids from wt (a) and H2A.B.3−/y (b) were indirectly immunostained with anti-Y12 antibody and counterstained with DAPI. Scale bar is 10 μm. White arrows show accumulation of Y12 signal in DAPI-depleted regions. The immunostaining pattern showed no difference in Y12 localization. (PDF 1236 kb) [file 13059_2019_1633_MOESM16_ESM.pdf]

Figure S10

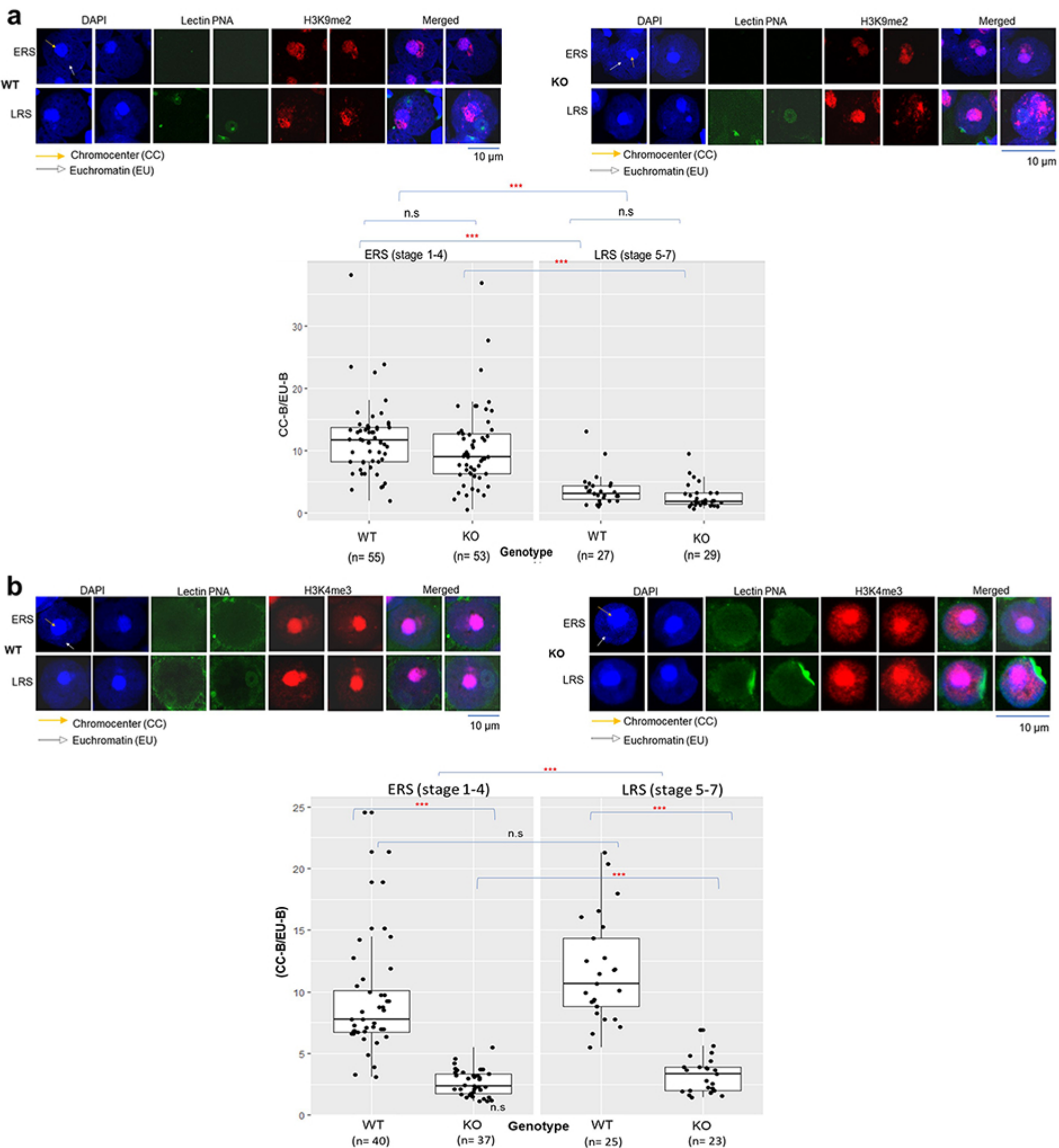

Supplement: Supplementary file 17 — Figure S10. H3K4me3 accumulates in euchromatin of H2A.B.3 KO round spermatids. Early round spermatids (ERS) and late round spermatids (LRS) were immunostained with H3K9me2 (panel a—red) and H3K4me3 (panel b—red) and Lectin PNA (green, a marker for acrosome to determine the stages of RS cells). DNA was co-stained with DAPI (blue). Scale bar is 10 μm. The quantification of H3K9me2 and H3K4me3 was based on the ratio of (CC − B)/(EU − B). *** ps0.001, ANOVA test. (PDF 1323 kb) [file 13059_2019_1633_MOESM17_ESM.pdf]

Figure S11

**a**

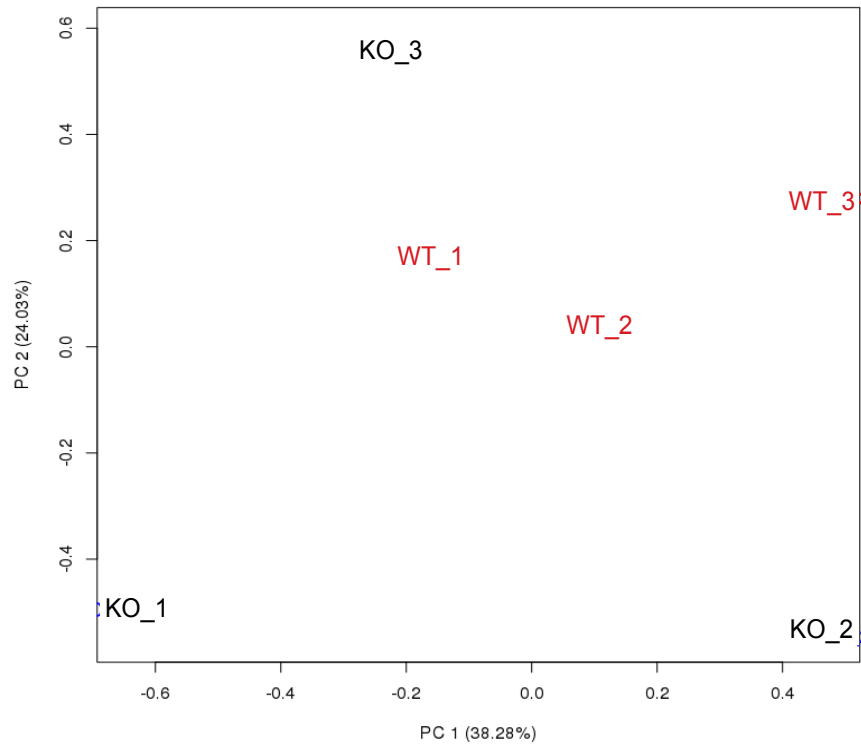

**b**

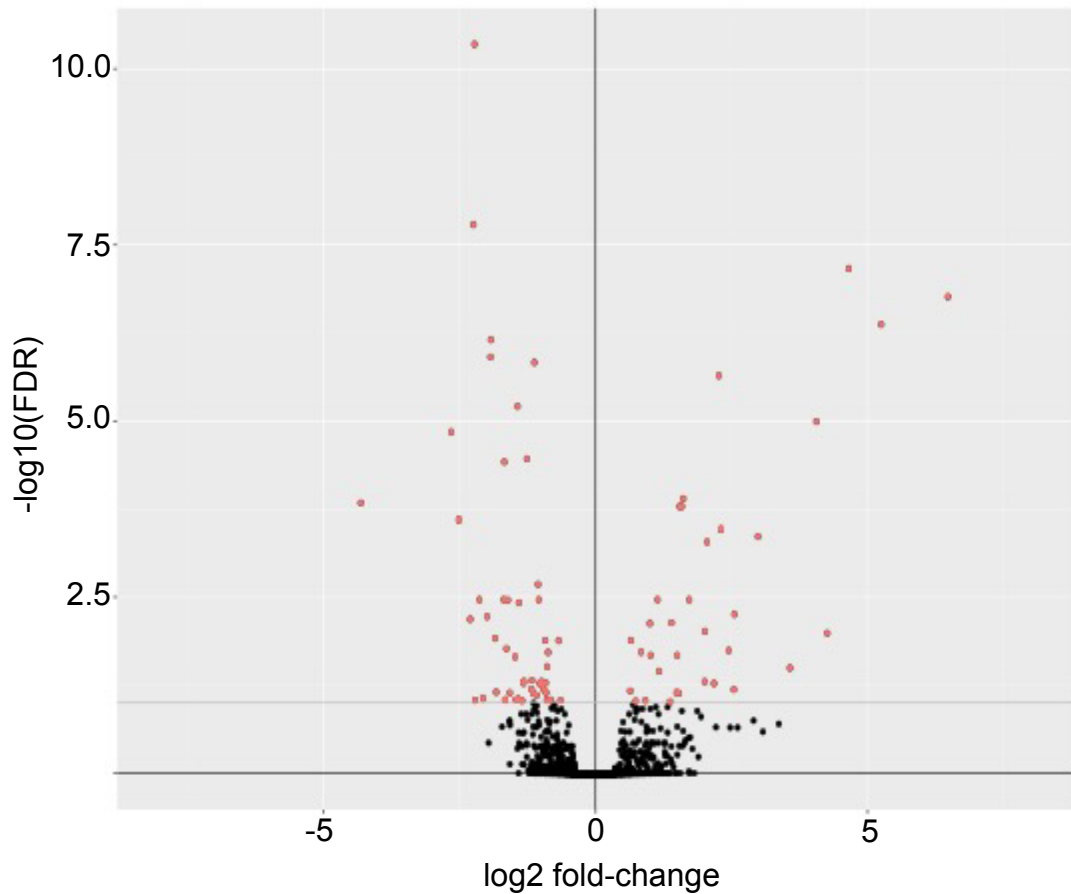

Supplement: Supplementary file 18 — Figure S11. Differentially expressed genes between H2A.B.3KO and wt round spermatids. (a) Principal component analysis of three sequenced H2A.B.3KO and wt mRNAseq libraries (each library combined mRNA from two wt or two H2A.B.3 KO mice). (b) Log2-changes in gene expression are plotted against significance (−log10-FDR). The plot illustrates in red those genes (Additional file 23: Table S11) whose expression is significantly altered in the absence of H2A.B.3. (PDF 201 kb) [file 13059_2019_1633_MOESM18_ESM.pdf]

Figure S12

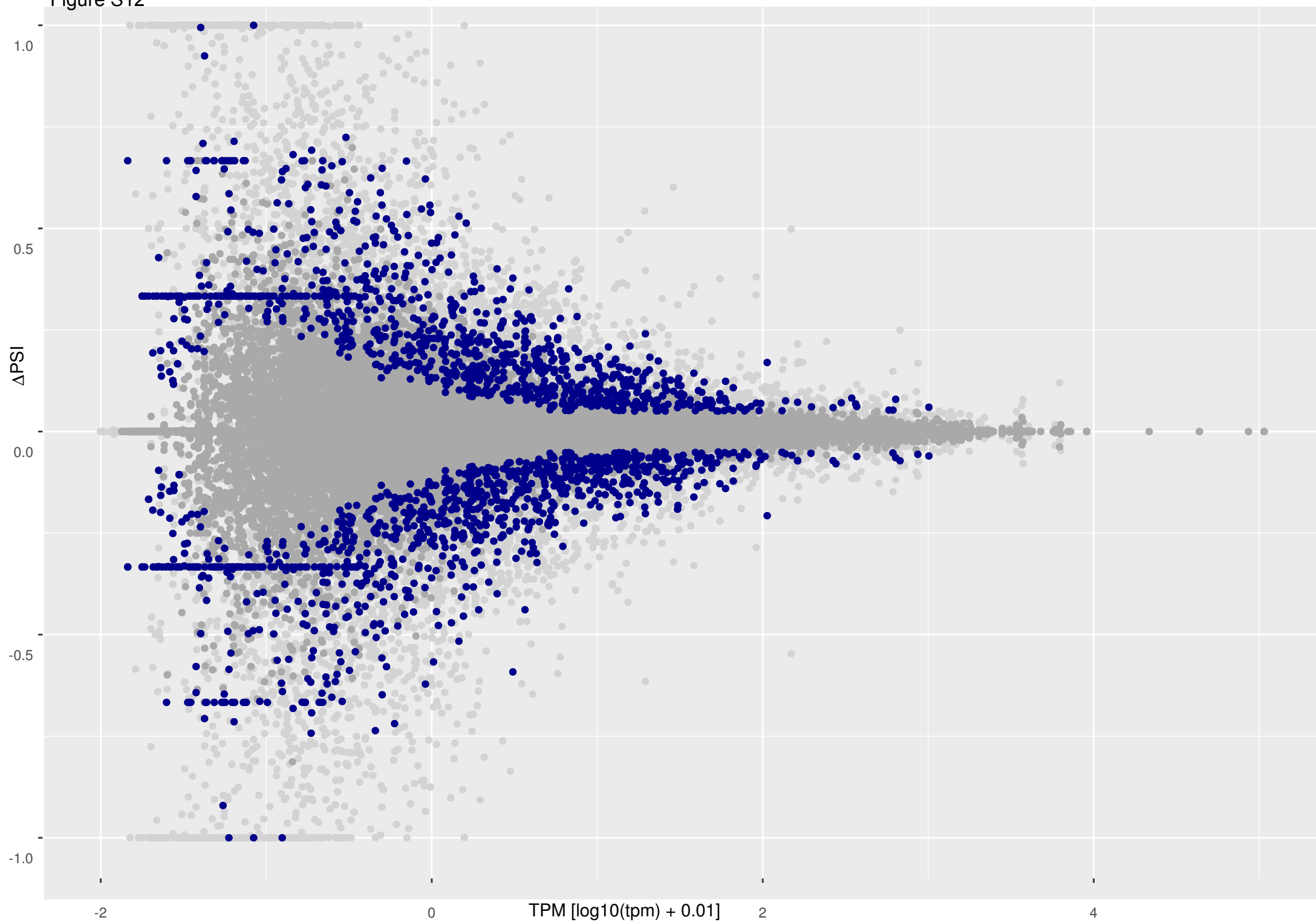

Supplement: Supplementary file 20 — Figure S12. Loss of H2A.B.3 affects pre-mRNA splicing. The analysis is based on SUPPA2. Illustrated is the distribution of altered splicing events by plotting the difference of percentage-spliced-in (PSI, the ratio between reads for included or excluded exons) versus transcript abundance of the transcripts associated with the splicing event (TPM) for two different conditions. The light gray dots correspond to (ΔPSI, TPM) values based on pairwise comparisons of WT versus H2A.B.3 KO replicates (i.e. three pairwise comparisons for the three biological replicates of WT versus KO) and TPM values averaged across replicates for each condition. The dark gray dots correspond to (ΔPSI, TPM) values after averaging PSI and TPM values across replicates for each condition. The dark gray dots show the difference in mean (averaged across replicates) PSI values and mean (averaged across replicates and conditions) TPM values. The blue dots represent events based on the replicate-averaged PSI values (the dark gray dots) that are statistically significant at an FDR of 5% (these numbers are shown in Table 2). (PDF 919 kb) [file 13059_2019_1633_MOESM20_ESM.pdf]

Figure S13

a

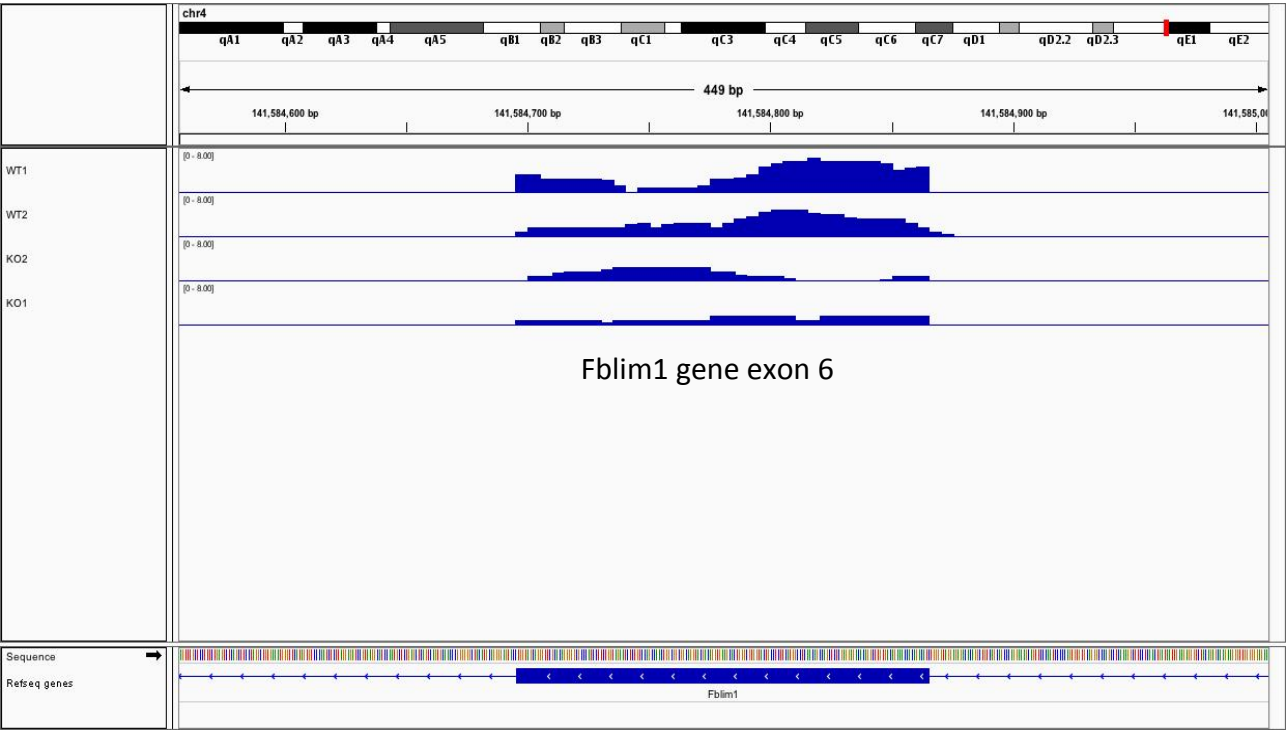

b

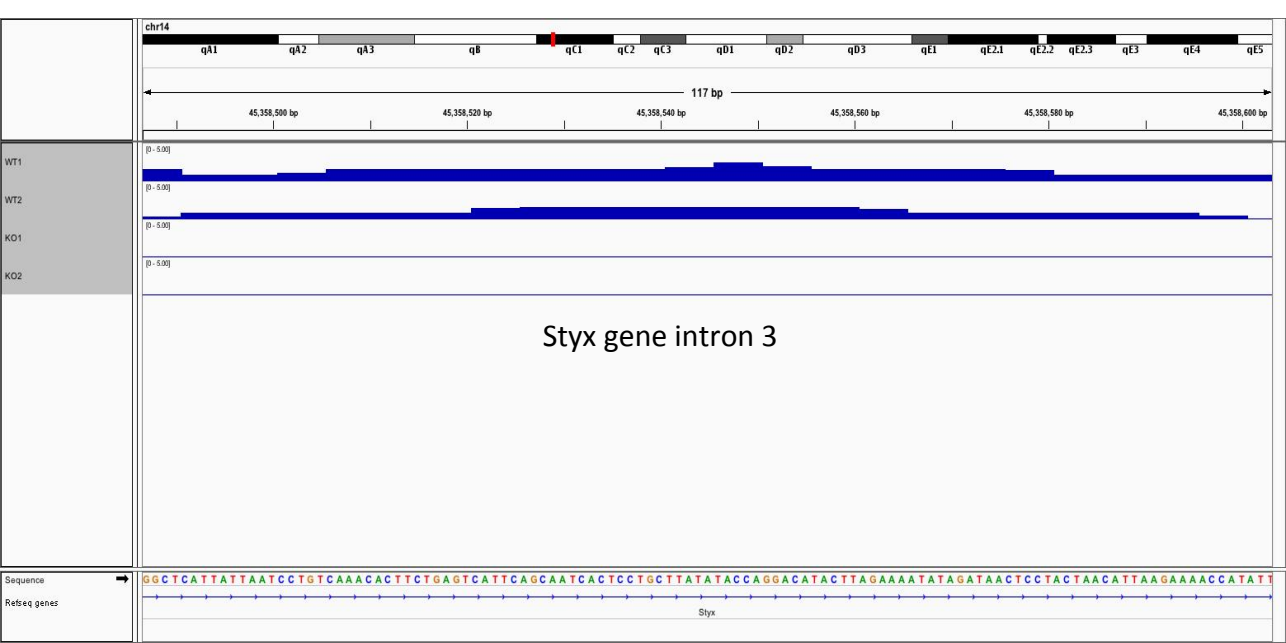

c

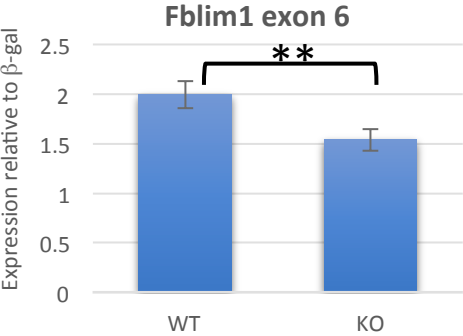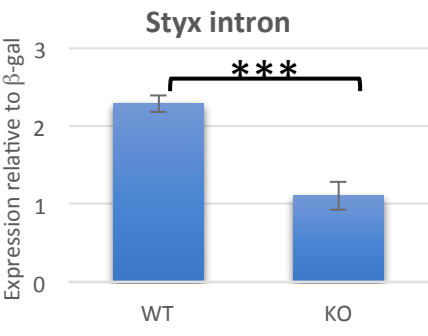

Figure S13 continued

d

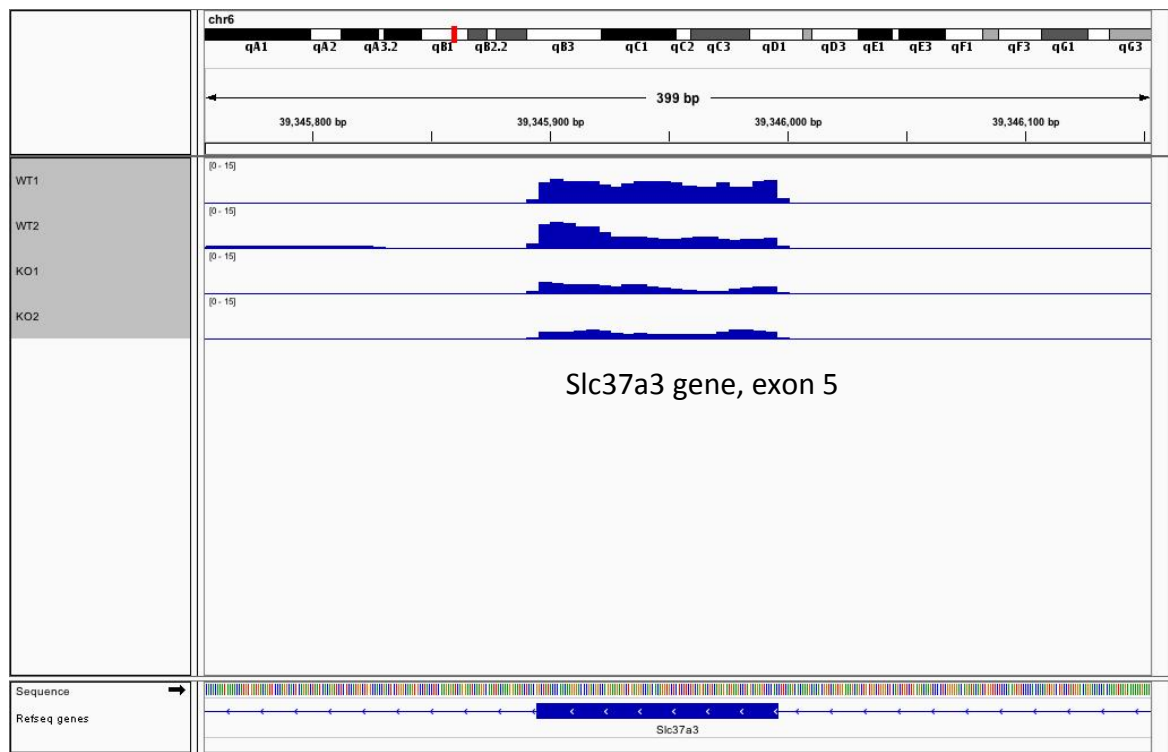

e

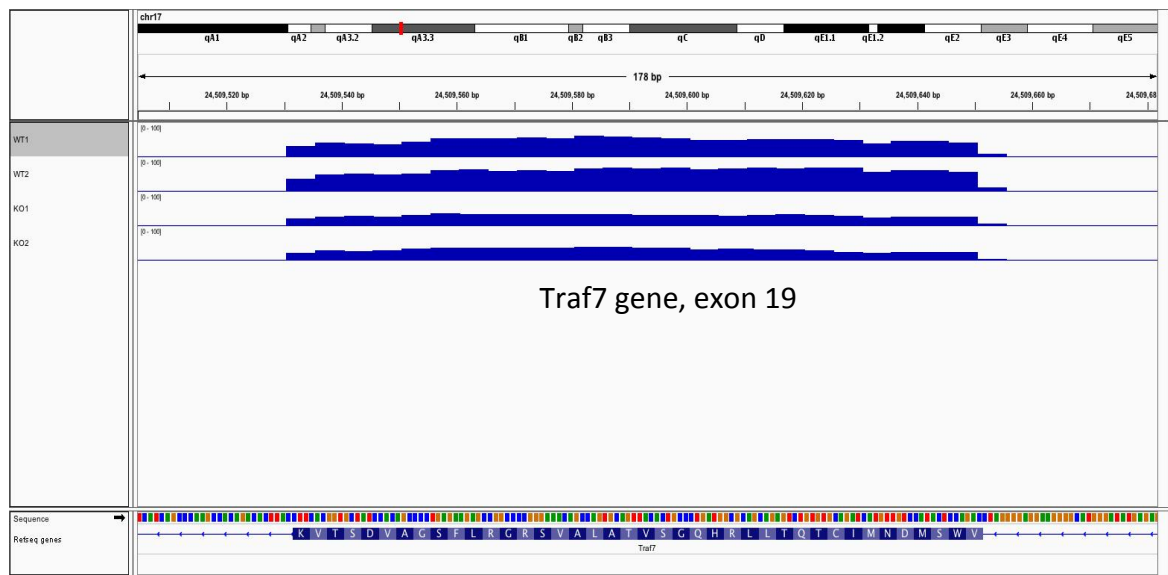

f

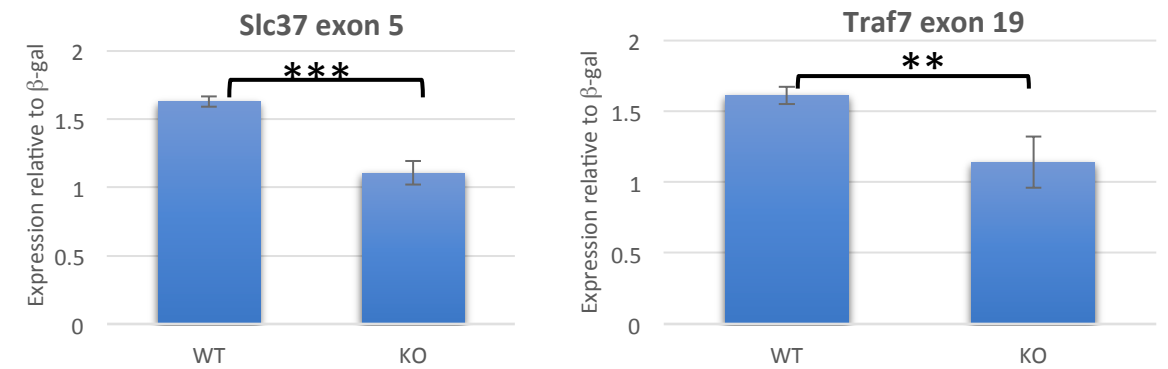

Supplement: Supplementary file 29 — Figure S13. Differential splicing events. The differential splicing events for specific genes between wild type and H2A.B.3 KO identified by SUPPA analysis were visualized in the IGV browser. Representative images are shown for Fblim1 exon 6 (a; differential transcript usage), Styx intron 3 (b; retained intron), Slc37a3 exon 5 (d; differential transcript usage) and Traf7 exon 19 (e; differential transcript usage). These individual examples of differential splicing events were confirmed by qPCR (c and f). Purified round spermatids from four biological replicas of wild type and H2A.B.3 KO mice were used. β-actin was used as the internal control. Error bars represent standard deviation. Student T-test was used to calculate p values (** p < 0.01; ***, p < 0.001). (PDF 419 kb) [file 13059_2019_1633_MOESM29_ESM.pdf]
