# Supplementary material for: Gene editing of the multi-copy H2A.B gene and its importance for fertility
Source: Genome Biol. 2019 Jan 31;20:23. doi: 10.1186/s13059-019-1633-3 (PMC6357441; doi:10.1186/s13059-019-1633-3)
Supplement: Supplementary file 3 — Table S2. List of TALENs designed to target H2A.B.3 genes. (PDF 51 kb) [file 13059_2019_1633_MOESM3_ESM.pdf]

Target genes	Design #	Targeted sequence
H2Afb3 (Group 1)	101406	ttCAGGTCGCCGCCACCGTcg
	101407	gtCGCCGCCACCGTCGCTcc
	101408	gtCGCCGCCACCGTCGCTCcc
	101409	ctCACAGCAAAGATTAGCTca
	101410	ctCACAGCAAAGATTAGCtc
	101412	ctCACAGCAAAGATTag
	101413	ctGAGCTAATCTTTGCTgt
	101415	ctAATCTTTGCTGTga
	101416	ctAATCTTTGCTGTGAgc
	101417	ctAACCTCCCTCAGATGCTgt
	101418	ctAACCTCCCTCAGATgc
	101419	ctCCGGGCACGGCTAACCTcc
H2Afb3, Gm14920, H2afb2 (Group 2)	101421	ctCCCGCACCTCCAGAGct
	101422	ctGTTCCACCAGGCTCACag
	101423	ctGCTGTTCCACCAGGCTca
	101424	ctGGTGGAACAGCATCTga
	101426	gtATCACTGAGCCTCCgg
	101427	gtTGCTGGAGCTTgc
	101429	gtACCTCTGCGTTcc

Table S2.
