# Supplementary material for: Gene editing of the multi-copy H2A.B gene and its importance for fertility
Source: Genome Biol. 2019 Jan 31;20:23. doi: 10.1186/s13059-019-1633-3 (PMC6357441; doi:10.1186/s13059-019-1633-3)
Supplement: Supplementary file 6 — Figure S4. TALEN-targeted DNA sequences. The alignment of H2A.B.3 genes and the pseudogene. TALENs specific to H2afb3 are in blue and those common for all H2A.B3 genes are in red. (PDF 58 kb) [file 13059_2019_1633_MOESM6_ESM.pdf]

Figure S4

```
                                               101408 CGCCGCCACCGTCGCT
                                               101407 CGCCGCCACCGTCG
                                                               101421 CCCGCACCTCCAGAG
H2Afb3      ATGCCAAGGAACAGGGAAAACTGTCTTCGAGAGTCTTCAGGTCGCCGCCACCGTCGCTCCCGCACCTCCAGAGCT
gm14920     ---------------------------------------------------------------------------
H2Afb2      -------------C--------------A-AG-----------A------A-A--A-----------------G-
Pseudo      ----------G--CA------A-------T-G-G--------T--A------T-T------:------------C

              CGATTAGAAACGACAC 101410
                 TTAGAAACGACAC 101412
H2Afb3      GAGCTAATCTTTGCTGTGAGCCTGGTGGAACAGCATCTGAGGGAGGTTAGCCGTGCCCGGAGGCTCAGTGATACG
gm14920     -----G--------A------------------------------A-----------T---------------T-
H2Afb2      -----G--------A----------------------------------------------------------T-
Pseudo      -----G------A-A------------------AG---------CC------A----T---------------T-
                           CACTCGGACCACCTTG 101422


H2Afb3      GTGCCCATCTTCCTGGCAGCCATCCTGGAGTCCCTCACCCGCAGGTTGCTGGAGCTTGCCGGCAATGAGGCCCAA
gm14920     -----------------------------------------------------------G---------------
H2Afb2      ------G---C-----T--------------T-------A-------------------G---------------
Pseudo      -----A-T-----------------------T-------TA----C-------------G---------A----G

H2Afb3      CGCAGAGGTACCGAGAGGCGCATCAACTCCTGAACTGCTGGACTTGGCTGTCTACAGCAATATGGAGCTAAGTGA
gm14920     ---------------------------------------------------------------------------
H2Afb2      ------------C------T-----------C---C-----------AG----------G---------------
Pseudo      -AG---------C---T-A----------------------------AG-----T---------C----------
```
