# Supplementary material for: Gene editing of the multi-copy H2A.B gene and its importance for fertility
Source: Genome Biol. 2019 Jan 31;20:23. doi: 10.1186/s13059-019-1633-3 (PMC6357441; doi:10.1186/s13059-019-1633-3)
Supplement: Supplementary file 8 — Table S3. The exome sequencing coverage. Three consecutive generations of mice from the NM4 H2A.B.3 KO colony were sequenced using paired-end sequencing with a 100 bp read length. (PDF 48 kb) [file 13059_2019_1633_MOESM8_ESM.pdf]

Sample ID	Total reads	Read length (bp)	Approximate coverage (x)
NM4-G1-28	226,900,000	100	613
NM4-G2-18	228,900,000	100	619
NM4-G3-32	129,800,000	100	351

Table S3. The exome sequencing coverage. 3 consecutive generations of mice from the NM4 H2A.B.3 KO colony were sequenced using paired-end sequencing with a 100bp read length.
