# Supplementary material for: Gene editing of the multi-copy H2A.B gene and its importance for fertility
Source: Genome Biol. 2019 Jan 31;20:23. doi: 10.1186/s13059-019-1633-3 (PMC6357441; doi:10.1186/s13059-019-1633-3)
Supplement: Supplementary file 9 — Figure S6. TALEN-induced deletions of the three H2A.B.3 genes. (PDF 92 kb) [file 13059_2019_1633_MOESM9_ESM.pdf]

Figure S6 TALEN-induced deletions of the three H2A.B.3 genes.

H2Afb3

WT FVB/NJArc CGCACCTCCAGAGCTGAGCTAATCTTTGCTGTGAGCCTGGTGGAACAGCATCTGAGGGAG
NM4 CGCACCTCCAGAGCTGAG - - - - - CTTTGCTGTGAGCCTGGTGGAACAGCATCTGAGGGAG Δ 5

Gm14920

WT FVB/NJArc CAGGTCGCCGCCACCGTCGCTCCCGCACCTCCAGAGCTGAGCTGATCTTTGCAGTGAGCC
NM4 CAGGTCGCCGCCACCGTCGCTCCCGCACCTCCAGAGCTGAG - - - - - - - - - - CAGTGAGCC Δ 10

H2Afb2

WT FVB/NJArc CAGGTCACCGCCAACATCACTCCCGCACCTCCAGAGGTGAGCTGATCTTTGCAGTGAGCC
NM4 CAGGTCACCGCCAACATCACTCCCGCACCTCCAGA- -

WT FVB/NJArc TGGTGGAACAGCATCTGAGGGAGGTTAGCCGTGCCCGGAGGCTCAGTGATATGGTGCCCGT
NM4 - GGCTCAGTGATATGGTGCCCGT Δ 64
