# Supplementary material for: Gene editing of the multi-copy H2A.B gene and its importance for fertility
Source: Genome Biol. 2019 Jan 31;20:23. doi: 10.1186/s13059-019-1633-3 (PMC6357441; doi:10.1186/s13059-019-1633-3)
Supplement: Supplementary file 10 — Table S4. Putative SNVs and Indels identified in H2A.B.3 KO mice using the mm10 mouse genome as a reference. (PDF 48 kb) [file 13059_2019_1633_MOESM10_ESM.pdf]

Sample ID	SNVs	Small deletions	Small insertions
NM4-G1-28	1,745,670	82,379	90,762
NM4-G2-18	1,768,957	81,956	90,005
NM4-G3-32	693,686	33,823	38,321

Table S4.
