# Supplementary material for: Gene editing of the multi-copy H2A.B gene and its importance for fertility
Source: Genome Biol. 2019 Jan 31;20:23. doi: 10.1186/s13059-019-1633-3 (PMC6357441; doi:10.1186/s13059-019-1633-3)
Supplement: Supplementary file 11 — Table S5. Putative SNVs and Indels identified in H2A.B.3 KO mice after applying the FVB/NJ strain filter. (PDF 48 kb) [file 13059_2019_1633_MOESM11_ESM.pdf]

Sample ID	SNVs	Small deletions	Small insertions
NM4-G1-28	21,256	3,889	4,378
NM4-G2-18	26,646	4,064	4,445
NM4-G3-32	13,417	1,589	2,032

Table S5.
