# Supplementary material for: Gene editing of the multi-copy H2A.B gene and its importance for fertility
Source: Genome Biol. 2019 Jan 31;20:23. doi: 10.1186/s13059-019-1633-3 (PMC6357441; doi:10.1186/s13059-019-1633-3)
Supplement: Supplementary file 12 — Table S6. Predicted putative homozygous and heterozygous polymorphic variants (off-target deletions) in three consecutive generations of H2A.B.3−/y mice. Exome data was referenced to mm10 genome, followed by filtering of FVB/N-specific variants and analyzed for genome polymorphism using Pindel tool. Homozygous polymorphisms: no polymorphic alleles (0/0), both alleles differ from reference (1/1), and both alleles differ from reference and from 1/1 (2/2). Heterozygous polymorphisms: one allele differs from the reference genome (0/1), one allele defers from the reference genome and from 0/1 (0/2), both alleles differ from the reference genome and from each other (1/2). No call, not assigned to any type. (PDF 54 kb) [file 13059_2019_1633_MOESM12_ESM.pdf]

Sample ID	Genotype							
	Homozygous			Heterozygous			No Call	Total
	0/0	1/1	2/2	0/1	0/2	1/2		
NM4-G1-28	131	732	2	388	2	27	111	1393
NM4-G2-18	127	727	3	392	3	25	116	1393
NM4-G3-32	219	527	8	306	4	15	314	1393

Table S6.
