# Supplementary material for: Gene editing of the multi-copy H2A.B gene and its importance for fertility
Source: Genome Biol. 2019 Jan 31;20:23. doi: 10.1186/s13059-019-1633-3 (PMC6357441; doi:10.1186/s13059-019-1633-3)
Supplement: Supplementary file 14 — Table S7. Nineteen common putative heterozygous polymorphisms identified in all three generations of H2A.B.3−/y mice. (PDF 72 kb) [file 13059_2019_1633_MOESM14_ESM.pdf]

#	Chromosome	Locus start	Locus end	Deletion size (bp)	Gene ID
1	1	171573961	171573965	4	ENSMUSG00000004709
2	1	173270636	173270648	12	ENSMUSG00000049605
3	1	173760152	173760189	38	ENSMUSG00000073489
4	12	115158545	115158549	5	ENSMUSG00000093894
5	12	115335195	115335199	5	ENSMUSG00000095197
6	12	115808522	115808526	21	ENSMUSG00000091087
7	12	115833994	115834007	14	ENSMUSG00000096020
8	17	23882468	23882474	7	ENSMUSG00000096445
9	17	35266213	35266218	6	ENSMUSG00000073411
10	17	36189406	36189415	10	ENSMUSG00000054128
11	17	48145527	48145531	5	ENSMUSG00000073386
12	2	119618224	119618250	27	ENSMUSG00000072980
13	3	7604075	7604079	5	ENSMUSG00000040329
14	4	41195246	41195333	88	ENSMUSG00000028433
15	4	42871823	42871856	34	ENSMUSG00000050141
16	5	113819659	113819689	31	ENSMUSG00000048163
17	7	8245023	8245027	5	ENSMUSG00000053720
18	7	47981089	47981093	5	ENSMUSG00000067173
19	X	95940663	95940678	16	ENSMUSG00000057421

Table S7.
