# Supplementary material for: Gene editing of the multi-copy H2A.B gene and its importance for fertility
Source: Genome Biol. 2019 Jan 31;20:23. doi: 10.1186/s13059-019-1633-3 (PMC6357441; doi:10.1186/s13059-019-1633-3)
Supplement: Supplementary file 30 — Table S17. PCR primers for genotyping of H2A.B.3 encoding genes in founders and the H2A.B3KO colonies. (PDF 53 kb) [file 13059_2019_1633_MOESM30_ESM.pdf]

H2A.B gene family	Sequence (5'-3')	Size of amplicon
H2A.Afb3	Fwd: CAGCAGAAAGCAGCCAAGTGG<br>Rev: GCAGGTCAGCCAAGAAGCA	440 bp
Gm14920	Fwd: GTTGGGCATTGGACTTGGAC<br>Rev: CAGCCAAGTCCAGCAGTTC	398 bp
H2Afb2	Fwd- CAGGTCAGCAGAGAGCAATT<br>Rev- CTCCATACTGCTGTAGACCT	374 bp

Table S17. PCR primers for genotyping of H2A.B.3 encoding genes in founders and the H2A.B3KO colonies.
