# Supplementary material for: Gene editing of the multi-copy H2A.B gene and its importance for fertility
Source: Genome Biol. 2019 Jan 31;20:23. doi: 10.1186/s13059-019-1633-3 (PMC6357441; doi:10.1186/s13059-019-1633-3)
Supplement: Supplementary file 31 — Table S18. List of primers to amplify 19 putative Indels from TALEN mutant mice. Symbols: ∆, deletion. (PDF 72 kb) [file 13059_2019_1633_MOESM31_ESM.pdf]

# Indel	Gene ID	Sequence (5'-3')	Expected product length (bp)
1	ENSMUSG00000004709 (Δ4)	F- AAGAAGACAGAACAGGGCTCAC<br>R- TAGCTCACATTGTCATCCTTGG	419
2	ENSMUSG00000049605 (Δ12)	F- GACAGTGTACACATTGGTCATT<br>R- TGGAGATGATGAGAATGTAAGA	458
3	ENSMUSG00000073489(Δ38)	F- TTAATGCCACATGATCAGTTAT<br>R- CCTAGACCATCTATTTTGGTTT	426
4	ENSMUSG00000093894 (Δ5)	F-AAGTCTACAAGCAAACCTTCTC<br>R-CATATACATGGGTGACAATGAC	452
5	ENSMUSG00000095197 (Δ5)	F-GCTGTATCTGAGCAAGTCTACA<br>R-ATACACTTGGGTGACAATGAC	464
6	ENSMUSG00000091087 (Δ21)	F-TAGATGGACATTTACAAAGCTG<br>R-TATCCTCACCTGTTCCAAAC	412
7	ENSMUSG00000096020 (Δ14)	F-TAGATGGACATTTACAAAGCTG<br>R-GCTACACCTTCACAAGCTACTA	402
8	ENSMUSG00000096445 (Δ6)	F-GTTGTGATGTCTTCTGTGCTC<br>R-GTACTCAGAACTGCCCACATA	405
9	ENSMUSG00000073411 (Δ5)	F-GGAAGGAGCAGAATTACACAT<br>R-GTTCAAGGAAGATCTTGACACT	468
10	ENSMUSG00000054128 (Δ10)	F-GTGCACTCCTGTGTCTTTTT<br>R-GCTGGACTACTACAACCTGAGT	451
11	ENSMUSG00000073386 (Δ5)	F-TTCTTGAGTCCTCTTCAACTCT<br>R-CTCTCTGACAGGAAGCATTTAT	413
12	ENSMUSG00000072980 (Δ27)	F-ACTCGGGAAAACAAAACAAAC<br>R-AAAGAAATATGGCGACTCTCT	418
13	ENSMUSG00000040329 (Δ5)	F-CAATGAAAGGGGTATAGTTCAT<br>R-AATTGTGAGAAGTTTCATTGTG	445
14	ENSMUSG00000028433 (Δ88)	F-CAGGGTCTAGTTTGTCCAGTAT<br>R-TTGCCATCTTTATCCTACAGAC	401
15	ENSMUSG00000050141 (Δ34)	F-AGGAGCTGACAGAGTCAATTT<br>R-GACGACTCCTGTGCTCAGAT	401
16	ENSMUSG00000048163 (Δ31)	F-CAGACGTTGTAAACAGAGTTTT<br>R-ACACAGTGGAGTCTAACCTCAG	409
17	ENSMUSG00000053720 (Δ5)	F-CAGACACTGAAGAACACTAGCA<br>R-TCACATTTGGAATTGTATTCAC	409
18	ENSMUSG00000067173 (Δ5)	F-CAGGTAGAGGCTCTTCATCA<br>R-ACCCGATGTTTTTGTTTATAGT	418
19	ENSMUSG00000057421 (Δ16)	F-TCTACATCATCACTTTCCTCAA<br>R-GCGAGTCTGTTCCATTTATACT	411

Table S18
